# Single Skyrmion Generation via a Vertical Nanocontact in a 2D Magnet-Based Heterostructure

**DOI:** 10.1021/acs.nanolett.2c01944

**Published:** 2022-11-18

**Authors:** Lukas Powalla, Max T. Birch, Kai Litzius, Sebastian Wintz, Frank Schulz, Markus Weigand, Tanja Scholz, Bettina V. Lotsch, Klaus Kern, Gisela Schütz, Marko Burghard

**Affiliations:** † Max Planck Institute for Solid State Research, Heisenbergstrasse 1, D-70569Stuttgart, Germany; ‡ Max Planck Institute for Intelligent Systems, Heisenbergstrasse 3, D-70569Stuttgart, Germany; § Helmholtz-Zentrum Berlin für Materialien und Energie GmbH, Hahn-Meitner-Platz 1, D-14109Berlin, Germany; ∥ University of Munich (LMU), Butenandtstraße 5-13 (Haus D), 81377München, Germany; ⊥ Institute de Physique, École Polytechnique Fédérale de Lausanne, CH-1015Lausanne, Switzerland

**Keywords:** magnetic skyrmions, 2D magnets, heterostructures, 2D spintronics, time-resolved X-ray microscopy

## Abstract

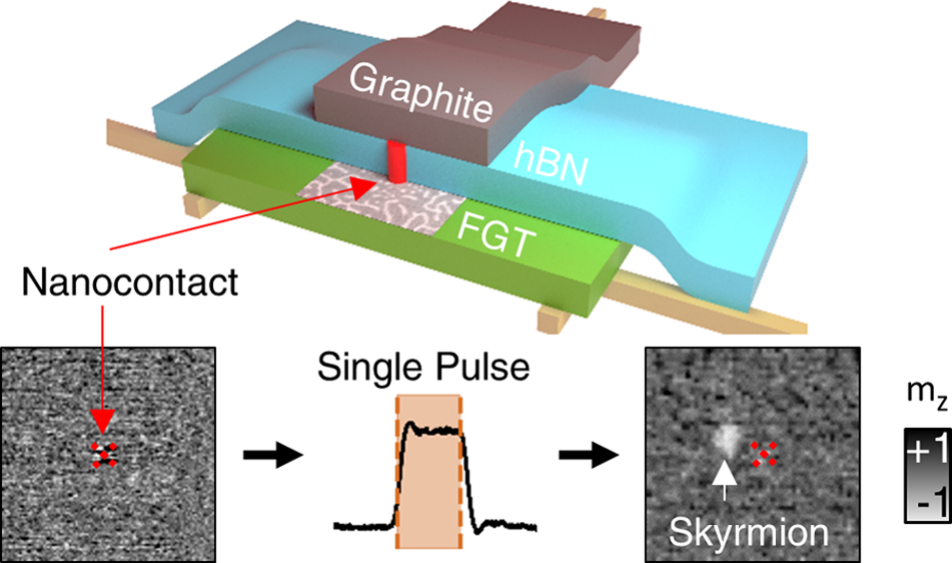

Skyrmions have been well studied in chiral magnets and magnetic thin films due to their potential application in practical devices. Recently, monochiral skyrmions have been observed in two-dimensional van der Waals magnets. Their atomically flat surfaces and capability to be stacked into heterostructures offer new prospects for skyrmion applications. However, the controlled local nucleation of skyrmions within these materials has yet to be realized. Here, we utilize real-space X-ray microscopy to investigate a heterostructure composed of the 2D ferromagnet Fe_3_GeTe_2_ (FGT), an insulating hexagonal boron nitride layer, and a graphite top electrode. Upon a stepwise increase of the voltage applied between the graphite and FGT, a vertically conducting pathway can be formed. This nanocontact allows the tunable creation of individual skyrmions via single nanosecond pulses of low current density. Furthermore, time-resolved magnetic imaging highlights the stability of the nanocontact, while our micromagnetic simulations reproduce the observed skyrmion nucleation process.

Magnetic skyrmions, nanoscale quasi-particles,^1,2^ are found in a range of materials stabilized by the Dzyaloshinskii–Moriya or dipole interaction^3,4^ and can exist in numerous forms, for example in Bloch-type, Neel-type, or antiskyrmion configurations, depending on the symmetry of the underlying host system.^5,6^ Because of their nanoscale size, the ability to manipulate them by charge and spin currents, and their topologically protected nature, skyrmions have attracted immense attention as potential information carriers for spintronic computing and storage applications beyond CMOS technology.^7−11^ These applications rely critically upon the controlled creation, annihilation, movement, and processing of skyrmions within suitable device architectures.^12^ Various approaches to create individual skyrmions in thin film systems have been experimentally demonstrated, specifically via local heating,^13−15^ current-induced spin–orbit torques,^14,16^ or electric fields.^17^ Among these, Joule heating effects have proven particularly useful to induce transitions between magnetic patterns in, for example, ferromagnet/heavy-metal heterostructures.^18−21^

2D vdW magnets,^22−25^ which have recently been established as novel skyrmion hosts,^26,27^ constitute a new material platform for investigating the manipulation of topological spin textures, for example, by magnetic fields^28^ or by structural geometry itself.^29^ In particular, the possibility to stack 2D materials into heterostructures provides numerous opportunities for material design and control^30,31^ such as the possibility that twisting two 2D sheets may give rise to Moiré skyrmions.^32^ An especially promising 2D magnet is Fe_3_GeTe_2_ (FGT), whose metallic character in combination with a sizable spin–orbit coupling (SOC)^33−36^ potentially enables the electrical manipulation of its skyrmion states.^27,37−40^ Depending on their thickness and iron content, exfoliated FGT sheets exhibit a Curie temperature, *T*_C_, between 150 and 220 K and a strong out-of-plane anisotropy.^33^ Recently, the current-driven motion of skyrmions, as well as the electrical generation of large skyrmion lattices, has been shown in FGT samples.^39^ However, the local and controlled creation of single skyrmions has yet to be demonstrated within a 2D magnet system.

In this work, we present a versatile method to locally nucleate individual skyrmions via nanosecond current pulses in an FGT-based vdW heterostructure. This nucleation is realized by current flowing via a vertical conduction channel between the FGT flake and the top graphite electrode. Utilizing X-ray magnetic circular dichroism (XMCD)-based microscopy, we identify the optimal parameters for controlling the number of nucleated skyrmions with single nanosecond current pulses. The survival of the conducting channel for more than 10^7^ pulses is a testament to its remarkable stability and suggests further applications in a wide range of 2D material-based devices.

The investigated vdW heterostructure consists of a mechanically exfoliated sheet of FGT (see Figure S1 for bulk magnetometry), which is separated from a top graphite layer by a 25 nm thick hBN sheet (see the Supporting Information). This structure was prepared on a Si_3_N_4_ membrane chip patterned with Au contact electrodes, enabling a voltage to be applied between the conductive FGT and the graphite, as schematically illustrated in Figure 1a. The FGT flake has different thickness regions ranging from 20 to 50 nm (see Figure S2 for AFM data), as seen in the scanning transmission X-ray microscopy (STXM) image in Figure 1b. Upon fabrication, the graphite was well-insulated from the FGT layer, as evidenced by a two-probe resistance measured between the two contact electrodes of 1 GΩ. However, we discovered that upon ramping up the applied voltage in 0.5 V steps, while limiting the current to a maximum of 10 μA, a single conduction channel formed locally between the FGT and graphite sheets after a certain voltage was reached. Across several devices, this voltage varied between 11 and 20 V, corresponding to a vertical electric field on the order of 1 × 10^9^ V m^–1^. After establishing this electrical connection, the two-probe resistance dropped abruptly to values between 50 and 100 kΩ, with the current limiter protecting the sample from further damage.

**Figure 1:**
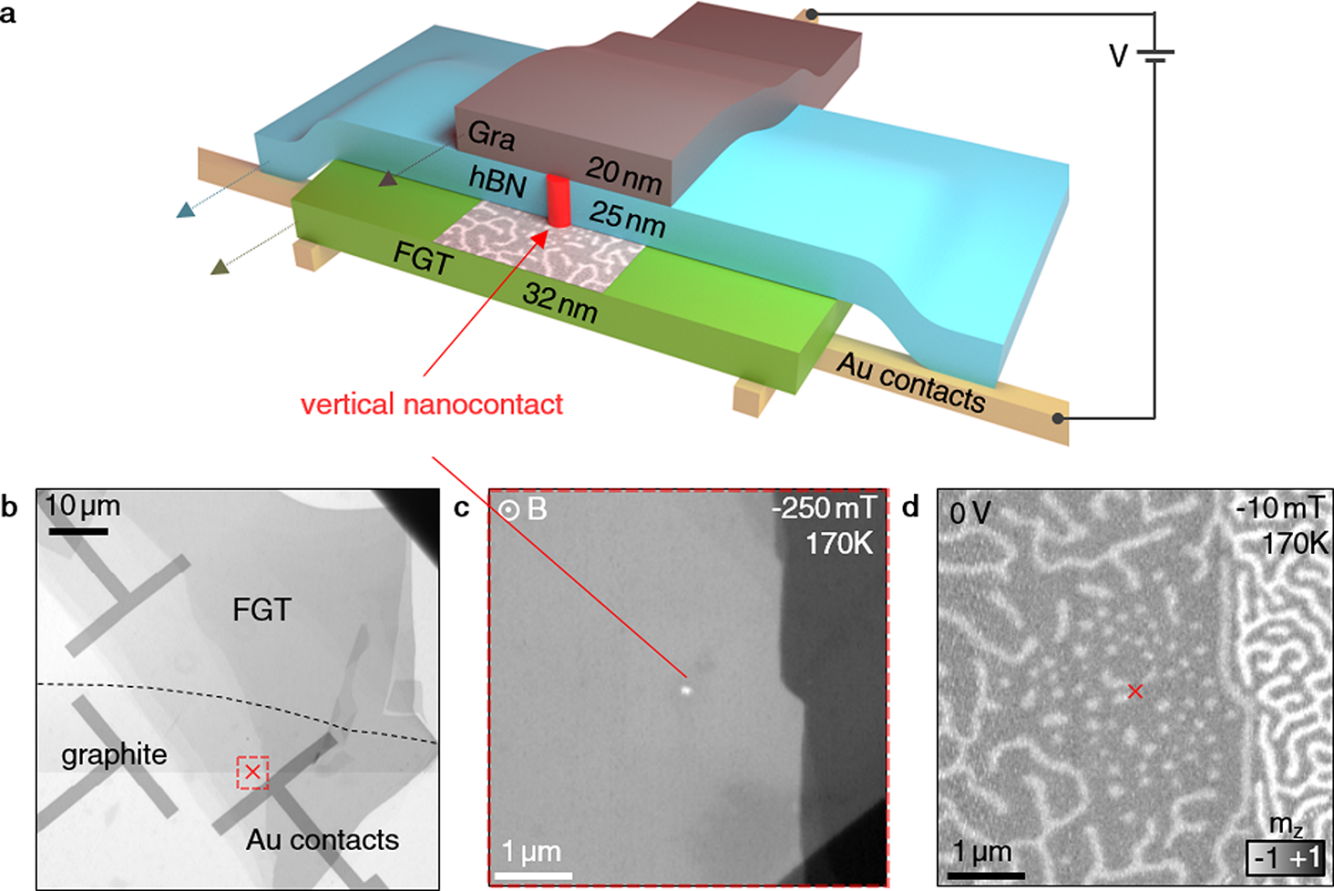
Fe_3_GeTe_2_ flake heterostructure device and vertical interlayer nanocontact. (a) Schematic illustration of the FGT/hBN/graphite heterostructure. The vertical nanocontact is marked by the red cylinder. (b) STXM overview image. The dotted line marks where the graphite sheet ends. (c) A magnified STXM image of the region around the nanocontact (white spot in the center), as shown by the red marked area in (b), exhibiting uniformly saturated magnetization at −250 mT. (d) Background-subtracted magnetic STXM image acquired after application of a static voltage of −3 V, revealing the nucleation of skyrmions around the nanocontact. The colormap is proportional to the out-of-plane component of the magnetization *m*_*z*_ (see Figure S4a for the electrical manipulation protocol).

The STXM image in Figure 1c shows an area of the heterostructure presenting a 50 nm hole in the FGT flake, as demonstrated by its comparably bright contrast. Through further imaging, we identified this hole region as the electrical contact between the FGT and graphite flakes. Because of the limited spatial resolution of STXM, the structure and detailed chemical composition of this nanocontact are not accessible. However, we speculate that either local heating or electrochemical reaction or migration resulted in a local breakthrough of the insulating hBN, establishing the electrical connection via migrated carbon or iron atoms, as illustrated in Figure 1a. Notably, the nanocontact formation was reproduced for a second sample following the same voltage procedure leading to similar observed behavior (see Figure S3) for which the FGT decomposition was not observed.

We investigated the possibility of utilizing this vertical conduction channel to locally influence the magnetic spin textures of FGT. STXM images were acquired before, during, and after applying both static and pulsed voltages, in various schemes (see Figure S4 for an overview). To achieve magnetic contrast, the energy of the X-rays was tuned to the L_3_ absorption edge of Fe, exploiting the effects of XMCD,^41^ which yields the out-of-plane magnetization component (*m*_*z*_), i.e., the domain structure in the FGT flake. For example, the image in Figure 1d shows the formation of skyrmions within a distance of 2 μm around the nanocontact, which was realized by applying −3 V for a few seconds (full image sequence in Figure S5). On the basis of previous Lorentz transmission electron microscopy (LTEM) measurements of similar FGT samples, we assume that the observed domain walls and skyrmions possess monochiral Néel-type domain walls.^39,42^ The exact configuration of the magnetic texture can be controlled by the magnitude and sequence of the applied voltages and the magnetic field (see Figures S6–S9).

The zero-voltage magnetic phase diagram of the present FGT flake is shown in Figure 2a, featuring the three known magnetic phases of FGT: the uniformly magnetized (UM), stripe domain (SD), and skyrmion (Sk) states, in agreement with previous observations.^42^ This diagram was determined by imaging of the nanocontact region of the FGT flake as a function of increasing applied perpendicular magnetic field after saturating the sample at −250 mT (see Figure S10 for representative data). Notably, at temperatures below 170 K, the sample exhibits monodomain switching behavior between oppositely oriented saturated states. However, the Joule heating induced by current flow through the nanocontact enables the rapid heating and cooling of the surrounding FGT flake. In this way, a sufficiently large applied current results in the formation of stripe domains or skyrmions at high temperature, which are subsequently frozen in as the sample rapidly cools back to lower temperatures, as indicated by the green arrow in Figure 2a. From a practical point of view, the choice of the magnetic field, initial temperature, and applied voltage can enable navigation of this magnetic phase diagram, resulting in targeted formation of the desired spin texture.

**Figure 2:**
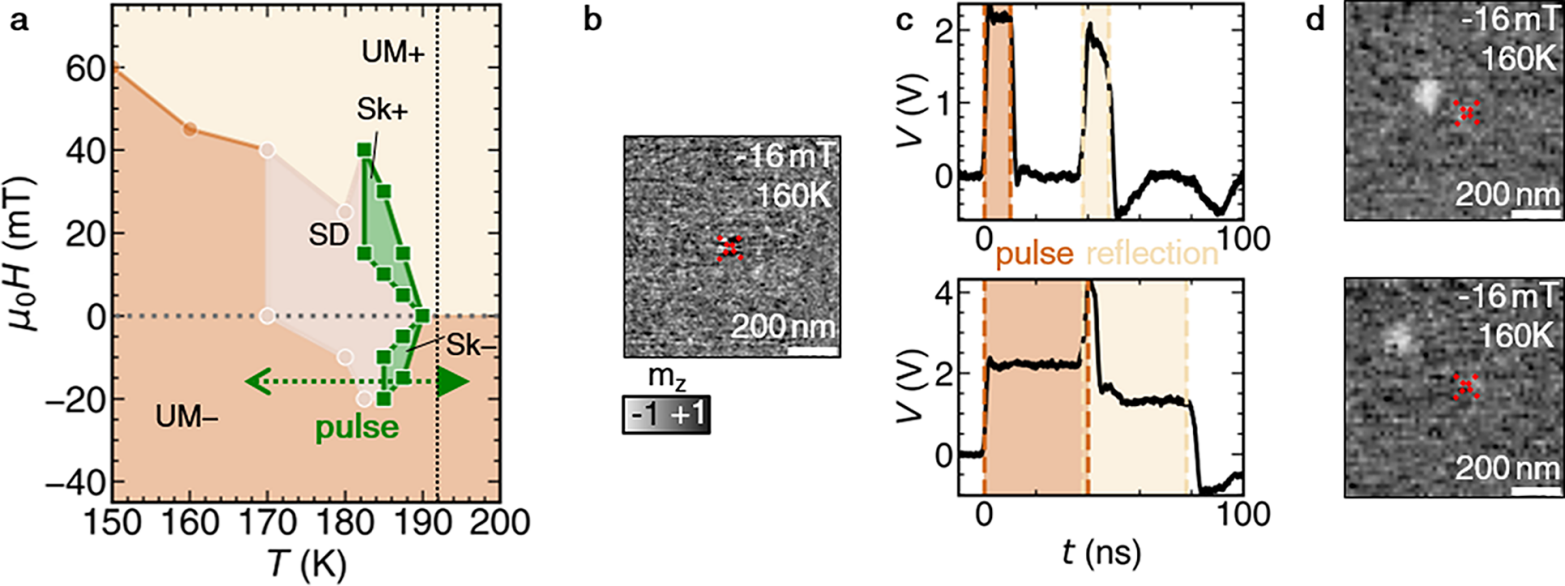
Magnetic phase diagram and single pulse, single skyrmion nucleation. (a) Magnetic phase diagram of the FGT flake region surrounding the nanocontact, with a thickness of about 32 nm, as determined from STXM measurements using a field sweep protocol. The phase diagram exhibits three different phases: uniformly magnetized (UM), stripe domain (SD), and skyrmion states (Sk). The green arrows indicate the effect of Joule heating (filled arrow) induced by the voltage pulse and subsequent field cooling (line arrow). (b–d) Generation of single skyrmions in the vicinity of a vertical nanocontact. The single skyrmions in both images of (d) were obtained in the vicinity of the nanocontact by single current pulses from a uniformly magnetized state, shown in (b). The colormap is proportional to the out-of-plane magnetization *m*_*z*_. In both cases, a pulse height of 2.25 V was utilized, but with different pulse lengths of 10 or 40 ns, as shown by the traces in (c) (see Figure S4c for the electrical manipulation protocol).

With this in mind, we investigated the possibility to nucleate skyrmions with single current pulses with a duration, Δ*t*, of a few nanoseconds and an amplitude, Δ*V*, of a few volts based upon the equivalent circuit in Figure S11. By exploring these parameters, we determined a regime of single-pulse, individual skyrmion formation. Figure 2b shows a uniformly magnetized state surrounding the nanocontact, initialized by saturating the sample with a magnetic field of −250 mT, followed by a field of −16 mT. In two examples, a single current pulse was applied to the nanocontact with Δ*V* = 2.25 V and a Δ*t* of either 10 or 40 ns, with the corresponding oscilloscope traces displayed in Figure 2c. The respective images recorded after these pulses are shown in Figure 2d. In each case, a single skyrmion with a diameter of about 100 nm was nucleated between 150 and 300 nm away from the nanocontact.

We performed further pulsed nucleation measurements for Δ*V* between 1.5 and 3.2 V and Δ*t* ranging from 2.5 to 40 ns. Example images acquired after pulses with Δ*V* = 2.5 and 2.95 V are shown in Figure 3a,b (see Figures S12–S15 for further pulse heights and lengths). The results reveal that the number and location of the nucleated skyrmions can be controlled by the tuning of Δ*V* and Δ*t*. The nucleation diagram in Figure 3c provides an overview of the number of nucleated skyrmions for each combination of pulse parameters. In the limit of long pulses, we observe that below a certain energetic threshold *E*_thr_ (corresponding to a threshold voltage *V*_thr_ labeled in Figure 3c) no skyrmions can be nucleated. We attribute this threshold to thermal effects facilitating the nucleation of skyrmions during the pulses.

**Figure 3:**
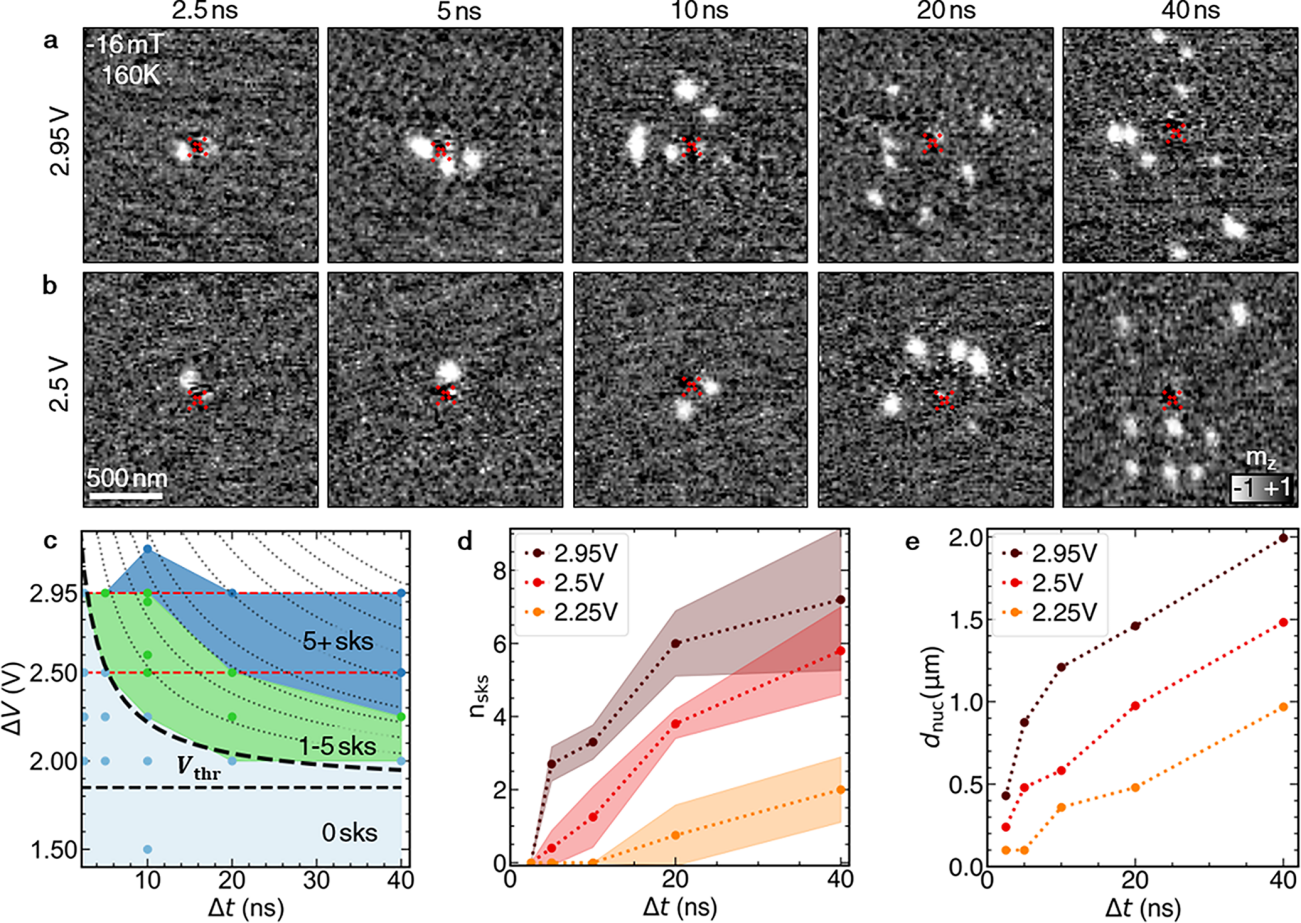
Control of the skyrmion nucleation by the pulse parameters. (a, b) Selection of two series of STXM images showing examples of skyrmion formation around the nanocontact (marked by the red cross), performed with two different voltage pulse heights, Δ*V*, of 2.95 V (a) and 2.5 V (b), and a range of pulse durations, Δ*t*. The colormap is proportional to the out-of-plane magnetization *m*_*z*_. (c) Number of skyrmions generated at *B* = −16 mT and *T* = 160 K as a function of Δ*t* and Δ*V*. The contours represent equipotential lines of the function , which we argue well describes the observed skyrmion nucleation by Joule heating effects. The black dashed line represents the threshold voltage above which at least one skyrmion can be generated. The red dashed lines highlight data presented in (a) and (b). (d) Average number of nucleated skyrmions (*n*_sks_) as a function of Δ*t* for three different voltage pulse heights. The colored regions indicate one standard deviation of the average. (e) Relationship between maximum nucleation radius *d*_nuc_ and Δ*t* for three different voltage pulse heights (see Figure S4c for the electrical manipulation protocol).

With the energy of the incoming pulse *E*_pulse_ and the energetic threshold *E*_thr_, the approximate skyrmion nucleation energy *E*_nuc_ can be calculated by
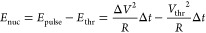
1Above the threshold, the term *E*_nuc_ determines the probability of skyrmion nucleation. This relation qualitatively explains the shape of the nucleation diagram, which is more clearly visualized by rearranging the function to . We have plotted equipotential contour lines (dotted) of this function in Figure 3c; the line that best describes the onset of single skyrmion nucleation (dashed) was realized with the following parameters: *E*_nuc_ = 0.3 nJ, *R* = 50 Ω, and *V*_thr_ = 1.85 V. Note that these parameters correspond to the total energy input into the circuit rather than the actual energy delivered to the sample (which is why the 50 Ω resistance of the oscilloscope is utilized). The above analysis assumes that Joule heating plays the major role in the skyrmion nucleation. However, we cannot rule out smaller contributions from spin-transfer torque (STT) as previously reported in the literature.^43,44^

The optimum conditions to induce an individual skyrmion can be deduced from the results shown in Figure 3d,e, where the number of induced skyrmions, *n*_sks_, and their maximum distance from the nanocontact, *d*_nuc_, are plotted as a function of Δ*t* for different Δ*V*. A larger pulse height is favorable for achieving single skyrmion nucleation, albeit this requires a pulse duration of just a few nanoseconds (Figure 3d). At the same time, a larger voltage leads to the resulting skyrmion being located farther away from the nanocontact (Figure 3e), thus enabling control over the distance of the nucleation from the nanocontact.

To shine a light on the mechanism of skyrmion nucleation, we performed dynamical STXM measurements using a pump–probe method. Repeated pulses were applied to the nanocontact with a duration of 60 ns, a separation time of 4 μs, and an amplitude of 2 V on top of a static voltage offset of 1 V (see the Supporting Information and Figure S16). The number of time channels utilized during the 4 μs observation time was 2001, resulting in a final time resolution of 2 ns. The subtraction of movies acquired with both circular X-ray polarizations resulted in a frame-by-frame view of the XMCD signal, and therefore *m*_*z*_, around the nanocontact during and after the pulse excitation. Individual frames acquired 14 and 62 ns after the start of the pulse are shown in Figures 4a and 4b, respectively, revealing a reduction in *m*_*z*_ which radially expands with a speed on the order of 10 m s^–1^. Figure 4c shows an average of frames acquired between 1000 and 4000 ns, where the system is close to equilibrium between the pulse excitations. This image features a ring-shaped area of reduced magnetic contrast centered around the nanocontact, which will be termed magnetic halo in the following.

**Figure 4:**
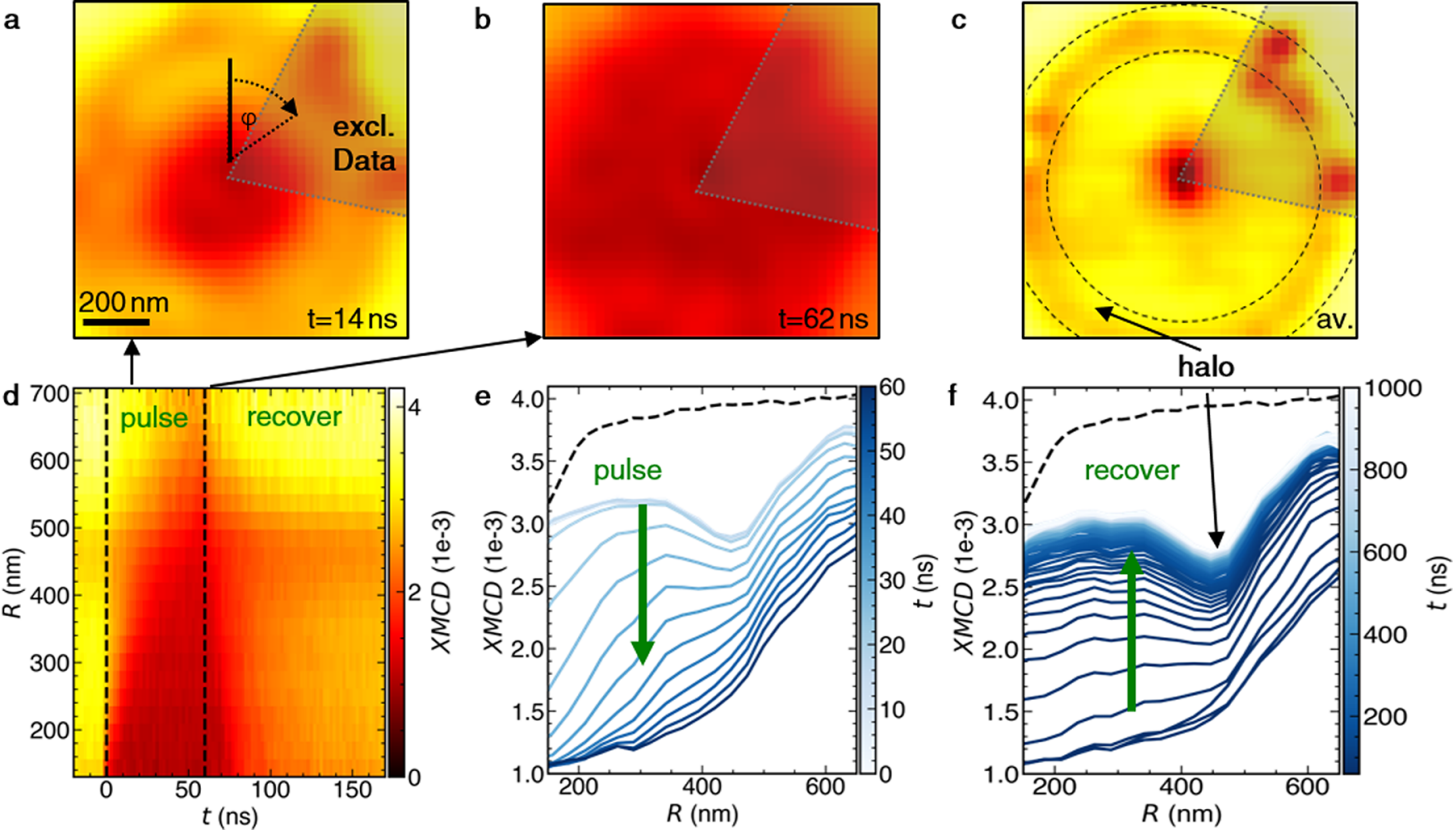
Dynamic measurements of the nucleation process. (a–c) XMCD images from a stroboscopic image series, recorded at different time delays after the nanosecond voltage pulse. All data were taken at *T* = 150 K, *B* = −16 mT, Δ*V* = 2 V, *V*_DC_ = 1 V, and Δ*t* = 60 ns. The static current *V*_DC_ was applied throughout the measurement. The shaded area marks excluded data, where we see enhanced skyrmion nucleation possibly by defects. (d) Temporal evolution of the XMCD signal, averaged over the azimuth angle, as a function of distance *R* from the nanocontact center. (e, f) Evolution of the XMCD signal averaged over the azimuth angle during (e) the applied pulse and (f) the subsequent signal recovery (see Figure S4g for the electrical manipulation protocol).

For each frame, we integrated over the azimuthal angle φ (see label in Figure 4a), resulting in the plot of the XMCD signal as a function of *R* and *t* in Figure 4d. Radial profiles of the XMCD signal in Figure 4d at various times are shown in Figure 4e,f. These plots show the temporal evolution of the XMCD signal during (Figure 4e) and after (Figure 4f) the applied pulse. The black dashed lines plot the XMCD signal in the absence of any current application. As is apparent from Figure 4f, the magnetization returns close to equilibrium within a few hundred nanoseconds, leaving behind the halo 500 nm away from the nanocontact center. Note that the halo can also be recognized at *t* = 0 before the pulse begins in Figure 4e. Figures S17 and S18 show the halo radius dependence on pulse duration and DC offset, indicating that the halo is not related to local material modifications but a tunable quantity.

With regard to the origin of the skyrmion nucleation process, two major mechanisms are most likely, namely thermally induced effects or current-driven STT, and there are three main pieces of evidence when distinguishing between these two scenarios. First, by utilizing the XMCD signal in the dynamic measurements, we estimated the temperature of the sample to be 181 K at a position 650 nm away from the contact, where no magnetic textures are nucleated (see Figure S19).^45^ According to the phase diagram in Figure 2a, this means that the region within the halo may well be at a high enough temperature to directly nucleate skyrmions. Second, the shape of the nucleation diagram in Figure 3c can be described solely by Joule heating. Third, our measurements indicated that the polarity of the applied pulse did not appear to have a significant effect on the number and position of the nucleated skyrmions (see Figure S20). Taken together, this strongly implies that the nucleation is primarily driven by Joule heating, rather than STT-based effects, although we cannot rule out a smaller contribution from the latter.

To explore the feasibility of this scenario, we performed micromagnetic simulations of the FGT system with a local radially decaying temperature gradient and current density under an applied out-of-plane field of 50 mT, as shown in Figure 5 (see the Supporting Information). Upon switching on the current and temperature gradient, we observed a nucleation and outward travel of magnetic domains, including short stripes and skyrmions, well-reproducing the observed experimental data.

**Figure 5:**
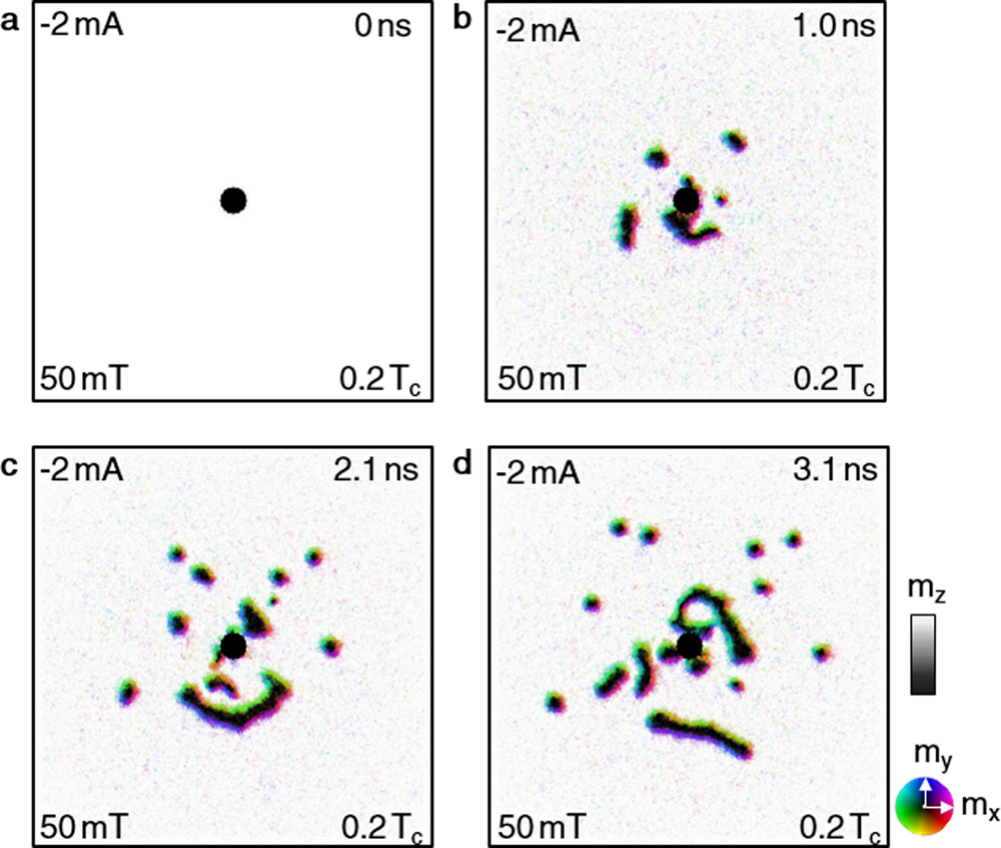
Micromagnetic smulations of the skyrmion formation via a nanocontact using the parameters for FGT as described in the Supporting Information. The applied field was 50 mT, and the applied current −2 mA. (a–d) show different times of the simulations, namely (a) 0 ns, (b) 1 ns, (c) 2.1 ns, and (d) 3.1 ns.

However, one feature that still demands explanation is the halo observed in the dynamic measurements with repeated pulsing, which was not reproduced directly in the simulations. It is important to note that the stroboscopic imaging method relies on averaging over many events per pixel (in this case, 188 pulses per pixel) and that upon discontinuing the excitation the halo vanishes, leaving only an arrangement of skyrmions. There appear two possible explanations for the observed halo. As a first option, the halo could be some kind of nonequilibrium transient domain, which manifests due to the interplay of the time-varying temperature and current gradient with the dipolar interaction. Alternatively, averaging of numerous skyrmion formation events during the pump–probe measurement could result in only a partial reduction of the XMCD signal. In this case, the halo would represent a specific radius of increased skyrmion observation probability. This could arise either because there is an increased nucleation probability directly at a specific radius^46,47^ or because the nucleated skyrmions are driven outward by either current-induced STT^39^ or the thermal gradient during the pulse event.^20^ As both the temperature and the current density fall off as an inverse of the distance from the nanocontact, the skyrmions could stop at a radius where the threshold for their motion is no longer met, resulting in the manifestation of the halo in the pump–probe measurement. Future theoretical studies could address further details of the dynamical evolution of the halo and its possible role in the skyrmion formation.

The present example of single skyrmion nucleation is just one possible application of the vertical nanocontact between layers within a 2D material heterostructure. Moreover, the stability of the contact after more than 10^7^ current pulses (as applied during the time-resolved imaging) demonstrates its potential usefulness for device application. This observation of such a localized nanocontact, with a diameter below 50 nm, may provide a natural explanation for why vdW heterostructures with a hBN gate insulator and graphite top gate may look intact by optical inspection, even though there is a significant gate-leakage current.

We emphasize that the present experiments did not aim to achieve control over the precise location of the nanocontact formation. This is likely determined by structural defects in the hBN, which are predetermined weak points where the electrical breakdown initiates. Possible candidates are atomic-scale defects such as lattice distortions, nonhexagonal bonding, impurities, or undesired doping,^48^ which occur in relatively high densities of  to  per layer. However, we speculate that defects on the microscopic scale like polydimethylsiloxane (PDMS) residues (strain), thickness fluctuations, wrinkles, and cracks^49−51^ could also be responsible for the position of the observed nanocontact formation. Purposeful positioning of the nanocontact may be feasible by deliberately implanting defects into the hBN, either with the aid of e-beam lithography or by focused ion beam, along the lines of other recently discovered lithography-free contacting methods of 2D materials.^52^ Furthermore, the implementation of an entire nanocontact array may enable the selective writing of grids of skyrmions, which could be exploited for neuromorphic computing applications.^53,54^

In conclusion, we have demonstrated that vertical conduction channels can be intentionally created in an FGT/hBN/graphite heterostructure while leaving the remainder of the sample entirely intact. The contact can sustain more than 10^7^ pulses with an approximate vertical current density of 8 × 10^11^ A m^–2^. Using scanning transmission X-ray microscopy, we demonstrated that such a nanocontact enables the local nucleation of individual skyrmions by single nanosecond voltage pulses. Beyond the present work on magnetic skyrmions, we envision that the lithography-free formation of the nanocontact and its inherent properties could inspire novel functions in vdW heterostructure devices, where the local vertical electrical connection of different 2D material layers may be desired.

## Data Availability

The data that support the findings of this study are available from the corresponding author upon request.

## Supporting Information Available

Sample fabrication, characterization measurements, description of methods and experimental protocols, micromagnetic simulations, additional data, and the reproduction of the nanocontact on a second device (PDF)
