# Supplementary material for: Single Skyrmion Generation via a Vertical Nanocontact in a 2D Magnet-Based Heterostructure
Source: Nano Lett. 2022 Nov 18;22(23):9236–43. doi: 10.1021/acs.nanolett.2c01944 (PMC9756335; doi:10.1021/acs.nanolett.2c01944)
Supplement: Supplementary file 1 — nl2c01944_si_001.pdf [file nl2c01944_si_001.pdf]

Supplementary Information: Single Skyrmion Generation via a Vertical Nanocontact in a 2D Magnet Based Heterostructure

Lukas Powalla,[*,†,‡] Max T. Birch,[¶,‡] Kai Litzius,[¶] Sebastian Wintz,[¶,§] Frank Schulz,[¶] Markus Weigand,[¶,§] Tanja Scholz,[†] Bettina V. Lotsch,[†,‖] Klaus Kern,[†,⊥] Gisela Schütz,[¶] and Marko Burghard[*,†]

†*Max Planck Institute for Solid State Research, Heisenbergstrasse 1, D-70569 Stuttgart, Germany*

‡*These authors contributed equally.*

¶*Max Planck Institute for Intelligent Systems, Heisenbergstrasse 3, D-70569 Stuttgart, Germany*

§*Helmholtz-Zentrum Berlin für Materialien und Energie GmbH, Hahn-Meitner-Platz 1, Berlin, D-14109 Germany*

‖*University of Munich (LMU), Butenandtstraße 5-13 (Haus D), 81377 München, Germany*

⊥*Institute de Physique, École Polytechnique Fédérale de Lausanne, CH-1015 Lausanne, Switzerland.*

E-mail: l.powalla@fkf.mpg.de; m.burghard@fkf.mpg.de

Supplementary Note 1: Sample Fabrication

The Fe_3GeTe_2 (FGT) single crystals were obtained by vapor phase growth. As the first step, polycrystalline Fe_3GeTe_2 was synthesized from the elements Fe, Ge and Te in a solid-state reaction. To this end, a stoichiometric mixture was vacuum-sealed in a quartz glass ampule, heated up to 625 °C with $100\,\mathrm{K\,h^{-1}}$, and then kept at this temperature for 60 h before cooling to room temperature with $100\,\mathrm{K\,h^{-1}}$.[1] Phase-purity of the gray powder was confirmed by X-ray diffraction. In the second step, single crystals of an average size of 1.5 x 1.5 x 0.5 $\mathrm{mm^3}$ were grown from the polycrystalline powder via a chemical vapor transport with iodine as transport agent in a temperature gradient from 750 °C to 700 °C for one week.[2] The composition of the single crystals was confirmed by energy dispersive X-ray spectroscopy (Tescan SEM Vega TS 5130 MM equipped with a silicon drift detector, Oxford) with a ratio of Fe : Ge : Te of 2.90(3) : 0.96(4) : 2. The gold contacts on the 100 nm thick SiN membrane were defined through e-beam lithography. To avoid strain in the final heterostructure, we evaporated only 3 nm of Ti and about 25 nm of Au. The FGT flake was selected and consecutively stamped using an all-dry viscoelastic transfer method. Initially, FGT bulk crystals were mechanically cleaved and exfoliated onto a PDMS stamp. Homogeneous flakes with a thickness of around 40 nm were selected by their optical contrast and stamped onto the predefined gold contacts. The FGT flakes were immediately capped by an hBN sheet with a thickness of about 20 nm. Finally, the gate-electrode was stamped on top of the hBN such that it makes electrical contact to the gold gate-electrode. The whole process was performed under ambient conditions, whereupon each side of the FGT flake was exposed for about 20 min to atmosphere.

Supplementary Note 2: Magnetometry and Thickness Evaluation by AFM and XMCD

Magnetometry measurements were carried out on the bulk single crystal with a Quantum Design MPMS3 vibrating sample magnetometer (see Supplementary Fig. S1a,b). The bulk FGT sample was aligned along the relevant crystal axis and fixed to a quartz glass rod using GE varnish. Temperature and applied magnetic field were controlled by a built-in helium cryostat. The bulk SQUID data indicates a bulk Curie temperature T_C of about 200 K and an out-of-plane easy axis. The energy utilised for the STXM images was optimized by acquiring circular positive spectra at positive magnetic saturation and negative magnetic saturation, which were used to calculate the XMCD spectra (see Supplementary Fig. S1c-f). As apparent from the large and sharp XMCD peak in Supplementary Fig. S1f, we used 707.6 eV for all images.

The structural integrity of the capped flakes and their thickness was confirmed by AFM measurements (Bruker Icon). However, the FGT thickness at the nanocontact location is difficult to determine by AFM since the step-like, cascade geometry of the FGT flake would require adding up the small steps that are smoothed out by the capping hBN layer. As alternative, we measure the AFM height of the largest step, and then use the XMCD signal calculated from two points of the STXM images in Supplementary Fig. S10 to calibrate the FGT-thickness at the nanocontact. The thick part of the FGT is d_{thick} =50 nm high, as apparent from Supplementary Fig. S2c,e. Assuming an approximated total oxidised FGT thickness of 10 nm, the averaged STXM counts in the thick and thin regions are: $\text{thick}_{+\text{B}} = 2925$, $\text{thick}_{-\text{B}} = 3300$, $\text{thin}_{+\text{B}} = 3953$, $\text{thin}_{-\text{B}} = 4224$. On this basis, it becomes possible to calculate the thickness of the thin region since the XMCD value determines the magnetic thickness:

$$\frac{t_1}{t_2} = \frac{\ln(\text{thin}_{+\text{B}}) - \ln(\text{thin}_{-\text{B}})}{\ln(\text{thick}_{+\text{B}}) - \ln(\text{thick}_{-\text{B}})}$$

Including the oxide layer, we receive a thickness for the region around the nanocontact of:

$$d_{\mathrm{thin}} \approx \frac{t_1}{t_2} \cdot (d_{\mathrm{thick}} - 10\,\mathrm{nm}) + 10\,\mathrm{nm} = 32\,\mathrm{nm}$$

Supplementary Note 3: Scanning transmission x-ray microscopy

Scanning transmission microscopy measurements were performed at the MAXYMUS end-station at the BESSY II electron storage ring operated by the Helmholtz-Zentrum Berlin für Materialien und Energie. With the sample mounted inside the microscope, cooling was achieved by a He cryostat, while the applied magnetic field was controlled by varying the configuration of four permanent magnets. The x-ray beam was focused to a ≈20 nm spot size using a Fresnel zone plate and an order separation aperture. The image was then acquired by scanning the sample pixel by pixel with the focused beam, with an x-ray energy of nominally 707.6 eV, using a piezoelectric motor stage. The resonant XMCD signal detected at the Fe L_3 edge at each point yielded an image including the magnetic structure. A single circular x-ray polarization was used to record most STXM images and compared to the saturated state for gaining pure magnetic contrast (differential magnetic contrast). Single photons were detected by an avalanche photo diode. We estimate that the spatial resolution of the STXM images. A Keithley 2450 was used to apply the static voltages.

Supplementary Note 4: Reproduction of nanocontact on a second sample

Supplementary Fig. S2 shows the reproduction of the nanocontact formation in a second

heterostructure. This was accomplished by increasing the applied voltage in a step-wise fashion, while limiting the current to 5 µA (at T =150 K). During the voltage ramp, the gate leakage remained below 50 pA until 11 V, where the nanocontact began to form. At an applied voltage of 13 V, the current limit (5 µA) was reached abruptly and the source measure unit consequently turned down the applied voltage to 7 V. By increasing the current limit to 20 µA, the resistance of the nanocontact could be further lowered, leading to an applied voltage of 5 V at 20 µA. This procedure can be iterated until the resistance of the nanocontact is in the desired range. In general, the resistance can be decreased from GΩ, where there is no conduction channel between the graphite and the FGT, to a vertical conducting channel with a resistance of MΩ down to 100 kΩ.

Supplementary Note 5: Different Pulsing Schemes

To explore the effect of passing a current through the nanocontact, we have used various (in total seven) different electrical manipulation/imaging protocols, as compiled in Supplementary Fig. S4. For Supplementary Fig. S4a-f, the acquisition time was between 1 ms and 50 ms per pixel (also depending on whether the line or point-by-point imaging mode was used at MAXYMUS). The first two protocols a) and b) involve electrical manipulation using static voltages. For protocol a), a series of static voltages is applied, then the static voltage turned to zero, followed by STXM imaging without any applied voltage. In protocol b) a static voltage is applied during the STXM imaging, enabling to detect spin textures which are, for instance, stabilised by a radial temperature gradient. Under these conditions, thermal equilibrium between the nanocontact and the surroundings should be well established. Protocols c) and d) involve the manipulation of the spin texture using nanosecond voltage pulses. In contrast to c), where no static current is applied during imaging, scheme d) applies a static voltage during image acquisition. Protocols e) and f) correspond to quasi-dynamical measurements, where each pixel is averaged over multiple (typically more than

10) nanosecond voltage pulses. In f), a static voltage offset is applied. Finally, protocol g) allows probing in a pump-probe fashion, with each pixel and each time delay Δt being recorded separately. Combining all pixels yields a video showing the temporal evolution of the effect of the voltage pulse (dynamics).

Supplementary Note 6: Influence of static voltages on the spin texture

Supplementary Fig. S5 shows an example of how applying a static voltage influences the spin texture around the nanocontact. Starting from a uniform magnetic state (panel a), the application of $|V| = 2.5\,\mathrm{V}$ induces the formation of radially arranged stripes (panel b). Note that the radial change in temperature manifests itself in the spacing and width of the magnetic stripes. Closer to the nanocontact, the domain width is smaller and the stripes are squeezed together. In panel c, the temperature within a couple of 100 nm is high enough that some skyrmions start to emerge. After turning off the voltage completely (panel d), skyrmions appear in vicinity to the nanocontact due to the higher local temperature there, whereas farther away, the sample is still in the stripe phase. Supplementary Fig. S6 shows another static voltage series (panels a-h) for 0 mT applied field, in qualitative agreement with the above described behavior. Supplementary Fig. S6 l) proves that the skyrmion nucleation is local and no other spin texture are created. Supplementary Figs. S7/S8 show the results of repeated spin texture nucleation for different voltage protocols as indicated. It can be seen that the four repetitions lead to similar spin textures, although they are not nucleated at the same spot indicating that pinning is not dominant. The stripe domains in Supplementary Fig. S7, observed after applying different static voltages between 3.9 V and 4.2 V for a few seconds and lowering the static voltage to 2.2 V during imaging, are arranged radially around the nanocontact. It can be seen that higher static voltages lead to larger nucleated structures. Supplementary Fig. S8 shows stripe domains arranged radially around

the nanocontact following the application of a DC voltage of $-3.9\,\mathrm{V}$ for a few seconds and lowering the DC voltage to $-2\,\mathrm{V}$ during imaging. Again, this protocol was repeated four times for different applied fields between 0 and -14 mT. We note that the offset voltage applied during imaging helps to stabilize the radially symmetric stripes. Comparison of images acquired during and after applying a voltage bias is made in Supplementary Fig. S9. Spin-orbit torque (SOT) can be excluded as origin of the radial arrangement of the stripe domains, since it would lead to magnetic stripes orthogonal to the current, in analogy to previous reports.[3] In general, heat gradients and STT both cause the formation of radially symmetry patterns around the nanocontact. A heat gradient modulates the magnetic anisotropy and saturation magnetisation, which in turn alters the stripe periodicity as a function of radius, thus favoring a radially symmetric pattern. In comparison, STT couples to a gradient of the magnetisation along the current flow, and thus leads to a vanishing torque and a restoring force in the radially symmetric stripe phase. While we are unable to separate the two effects in the transient formation state, for the stability of the final spin texture the thermal effect should be most relevant, as switching off the DC current partially destroys the stripes (see Supplementary Fig. S9), whereas STT-stabilized stripes are expected to only freeze in position.

Supplementary Note 7: Single nanosecond pulses

The equivalent circuit diagram of the setup used for the skyrmion generation is depicted in Supplementary Fig. S11. The different protocols are summarized in Supplementary Fig. S4c. For the nanosecond voltage pulse manipulation, we reset the magnetic state, apply one pulse with specific properties $(\Delta V, \Delta t)$ and record an STXM image afterwards. Due to the statistical nature of the nucleation, we repeated this four to five times for every combination of parameters. Supplementary Fig. S12 shows the full data set for a pulse height of $2.25\,\mathrm{V}$ and all pulse lengths. Supplementary Fig. S13 and S14 show more repetitions of the data

shown in the main text (Figure 3). Supplementary Fig. S15 provides additional data with various parameters. The averages values extracted from Supplementary Fig. S12-SS15 can be found in table S0.

Supplementary Table. S0: This table includes the average generated number of skyrmions dependent on the pulse lengths Δt and ΔV

ΔV(V)	2.5 ns	5 ns	10 ns	20 ns	40 ns
2	0	0	0	0	0.4
2.25	0	0	0	0.75	2
2.5	0	0.4	1.25	3.8	5.8
2.95	0	2.7	3.3	6	7.2
1.5	-	-	0	-	-
2.3	-	-	0	-	-
2.6	-	-	1.25	-	-
2.9	-	-	1	-	-
3.2	-	-	5.5	-	-

Supplementary Note 8: Time resolved XMCD

Time-resolved STXM was used to stroboscopically image the magnetization dynamics of the sample in an electric pump and x-ray probe fashion. This method utilizes the specific time structure of the incident x-ray flashes, which consists of pulses with a 2 ns repetition rate and ≈100 ps effective pulse length (x-ray probe). Each incoming probe event (photon transmitted or not transmitted through the sample) is routed after every pulse to a periodic counting register of a field-programmable gate array. Here, the number of registers (Q) sets the maximum stroboscopic observation period ($Q{\cdot}2\,\mathrm{ns}$). The sample is excited via the application of voltage pulses of length Δt and height ΔV to the gate electrode (electric pump) for which the pulses are synchronized to the time structure of the x-ray probe. Here the number of electric pulse excitations(S) within the maximum observation period sets the nominal time resolution ($2/S$ ns) and the stroboscopic observation time ($Q/S{\cdot}2\,\mathrm{ns}$). The incident/reflected pulse excitations were monitored on an oscilloscope via a −20dB pick-off tee at the input. The output of the sample was terminated to ground. The current density

that was applied during a pulse can be approximated using the measured voltage pulse height U_p and the reflected pulse height U_R at Oscilloscope at the bias-T of the pulsed setup (compare Supplementary Fig. S11, exemplary pulse at figure Fig. 1c).

$$\begin{aligned} U_\mathrm{L} &= U_\mathrm{p} - U_\mathrm{R} \\ U_\mathrm{T} &= U_\mathrm{L}\left(\frac{50\Omega}{R_\mathrm{p}+50\Omega}\right) \\ U_\mathrm{p} &= U_\mathrm{R} + U_\mathrm{T} \end{aligned} \tag{1}$$

Using this three equations,[4,5] we can simplify the dynamical resistance to be: $R_\mathrm{p} = 100\Omega\frac{U_\mathrm{R}}{U_\mathrm{p}-U_\mathrm{R}}$. Using one exemplary pulse with $U_\mathrm{p} = 2.2\,\mathrm{V}$ and $U_\mathrm{R} = 2.05\,\mathrm{V}$, we get an approximate value of $R_\mathrm{p} = 1360\Omega$. The current through the contact can be expressed via $I = \frac{U_\mathrm{p}+U_\mathrm{R}}{R_\mathrm{p}+50\Omega} \approx 3\,\mathrm{mA}$. At a radius of 400 nm away from the contact, assuming a FGT thickness of 32 nm, this leads to $j_1 = \frac{I}{2\pi Rd} \approx 4\cdot 10^{10}\frac{A}{m^2}$. The current density through the nanocontact itself under the assumption of a diameter of $2r$ =50 nm could be approximated by $j_2 = \frac{I}{2\pi r^2} \approx 8\cdot 10^{11}\frac{A}{m^2}$

Supplementary Note 9: Quasidynamical experiments

Interestingly, a halo can be observed not only in time-resolved measurements (Supplementary Fig. S4g), but also through static imaging while applying the pulsed measurements (Supplementary Fig. S4e,f). While the quasidynamical measurement scheme is unable to resolve time-dependent phenomena, it nonetheless reveals the temporal average of nonequilibrium physics.

Even the dependencies of the halo on parameters such as the pulse length or the voltage offset can be extracted. Because time-resolved experiments substantially require more time than quasidynamical images (approximately 1 hour versus 5 minutes), we took advantage of the quasidynamical method to extract the halo dependency on $\Delta t, V_\mathrm{static}$ (see Supplementary

Fig. S17,S18).

Supplementary Note 10: Temperature estimation using dynamical measurements

In general, the XMCD signal is directly proportional to the average out-of-plane magnetization of the sample. In the dynamical measurements, temperature as well as the presence of spin textures can decrease the measured magnetization. Away from the skyrmion nucleation region, it is plausible to assume that the change in magnetic contrast is only due to the temperature change. On this basis, we have used the equations and critical exponents α, β reported in reference[6] to describe the saturation magnetization of FGT ($\alpha = 0.34, \beta = 2.87$).

$$M(T) = M_0 \left(1 - \left(\frac{T}{T_\mathrm{c}}\right)^{2.87}\right)^{0.34} \tag{2}$$

$$T(M) = T_\mathrm{c} \left(1 - \left(\frac{M}{M_0}\right)^{\frac{1}{0.34}}\right)^{\frac{1}{2.87}} \tag{3}$$

This yielded a temperature of 184.2 K (180.7 K) at 600 nm (650 nm) away from nanocontact (based upon the 2 V pulse at 150 K in manuscript Fig. 4), as shown in Supplementary Fig. S19 and summarised in the following:

$$T\left(M(150\,\mathrm{K}) \cdot \frac{2.7}{4}\right) = 180.7\,\mathrm{K} \tag{4}$$

$$T\left(M(150\,\mathrm{K}) \cdot \frac{2.4}{4}\right) = 184.2\,\mathrm{K} \tag{5}$$

We used a Curie Temperature of $T_\mathrm{c} = 192\,\mathrm{K}$ leading to $M(150\,\mathrm{K}) = 0.79M_0$. The obtained temperature increase is consistent with the phase diagram in Fig. 2a. In particular,650 nm away from the nanocontact the temperature rises from 150 K to 180.7 K, no spin textures are nucleated, as the temperature remains below the expected nucleation temperature of 182 K.

In contrast, the temperature reached at 600 nm is high enough for skyrmion nucleation.

Supplementary Note 11: Micromagnetic Simulations

Simulations were performed in the MicroMagnum framework with custom extensions for temperature fluctuations and temperature dependent material parameters following the usual micromagnetic implementation. The simulated area was chosen as 600x600x20nm^3 discretized in a mesh of 300x300x1 cells. The current was included as a Zhang-Li Spin Transfer Torque (STT) caused by a radially symmetric current distribution, pointing away from the center of the nanocontact. The current density was calculated by assuming a constant current I_0 flowing through the nanocontact and then symmetrically spreading out into the magnetic material. This results in a current density of $j = I_0/(t2\pi r)$ at the distance r from the center of the contact (t being the thickness of the material). For the STT we assumed a $P = 1$ and $\xi = 0.05$. The temperature was simulated as a $(1 - r^2)$ dependence to account for the clamped temperature at the edges of the sample. Further simulations showed that a temperature dependence of r^{-1} leads to qualitative similar results. The temperature profile was applied in radially symmetric fashion and caused thermal field fluctuations and a change of material parameters following the equations

$$M_{\mathrm{S}}(T) = M_{\mathrm{S},0} \cdot \left(1 - \left(\frac{T}{T_{\mathrm{c}}}\right)^{2.87}\right)^{0.34}, \tag{6}$$

$$K_{\mathrm{u}} = K_{\mathrm{D}} + K_{\mathrm{eff}} = 0.5\mu_0 M_{\mathrm{S}}(T)^2 + 0.5 M_{\mathrm{S}}(T) H_{\mathrm{K}}. \tag{7}$$

H_K was set to 400mT, and M_S to 100kA/m. The magnetic damping was set to 0.1, the exchange to 0.7pJ/m and the DMI to 0.14mJ/m^2. Standing spin waves due to the limited box size were prevented by clamping the outermost spins on the edges of the simulated area with a strong field of 10T. The simulations were initialized with a homogeneously in out-of-plane direction, relaxed and then moved to a micromagnetic solver including temperature

effects.

Spplementary Figures

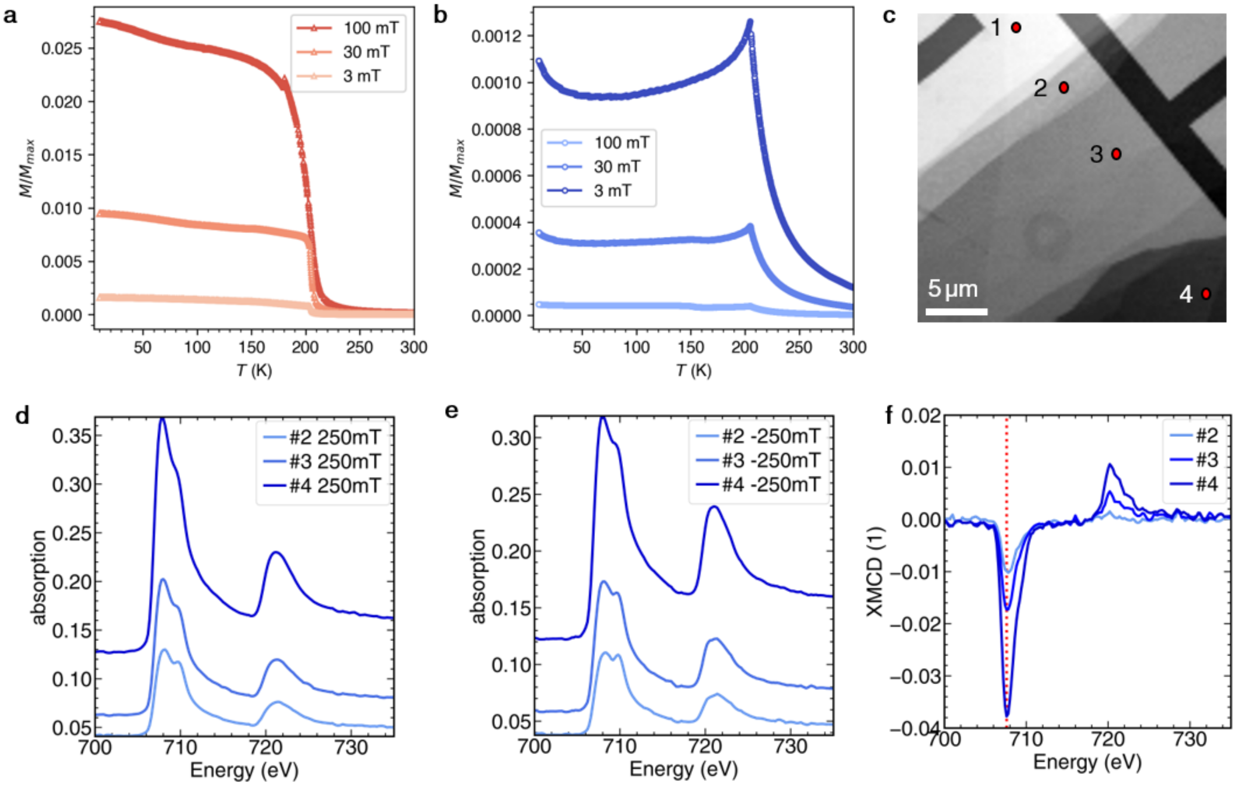

Supplementary Fig. S1: Magnetometry data of bulk Fe_3GeTe_2 and locally resolved XMCD spectra of the FGT flake. Normalized magnetization of the synthesized FGT crystal in (**a**) along c axis, and (**b**) in the a-b plane, in each case under three different magnetic fields. (**c**) STXM image of the flake with the measurement spots indicated. X-ray absorption for (**d**) c+ with 250 mT and (**e**) c+ with −250 mT, and (**f**) X-ray magnetic circular dichroism spectrum at the three different spots. The red dotted line indicates 707.6 eV.

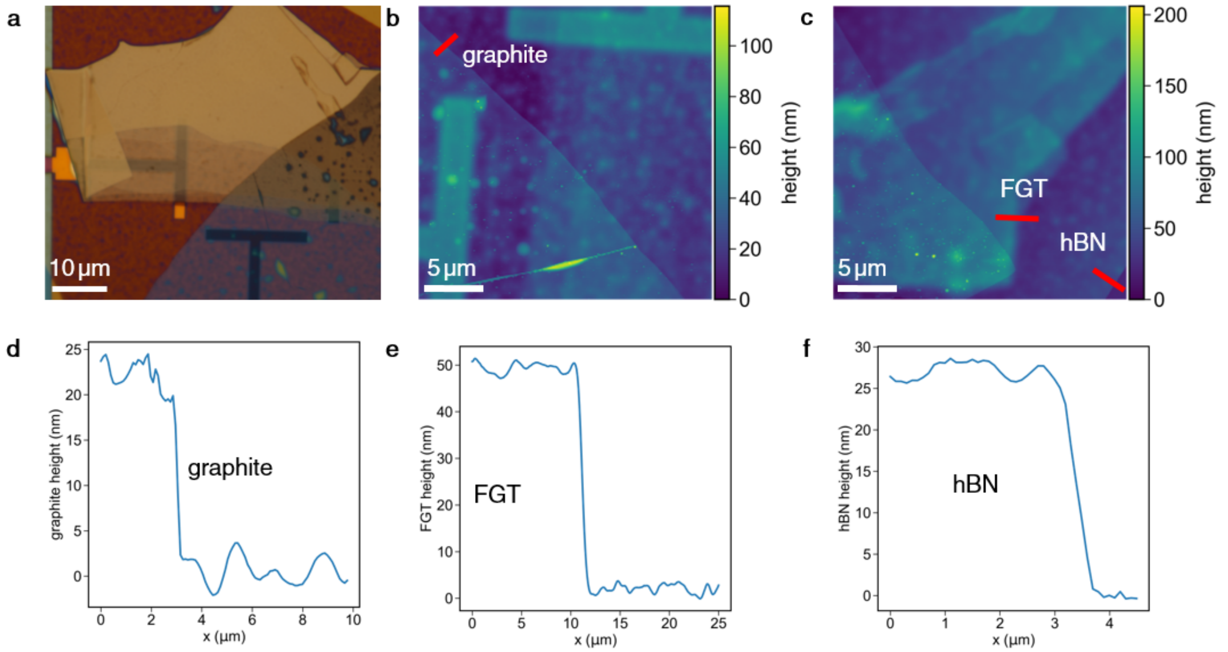

Supplementary Fig. S2: Optical micrograph and AFM characterisation of the main sample. (**a**) Optical image of the investigated heterostructure. (**b,c**) AFM images of two different regions on the sample. From the corresponding height profiles, we determined (**d**) a graphite thickness of 20 nm, (**e**) a maximal FGT thickness of 50 nm, and (**f**) a hBN thickness of 25 nm

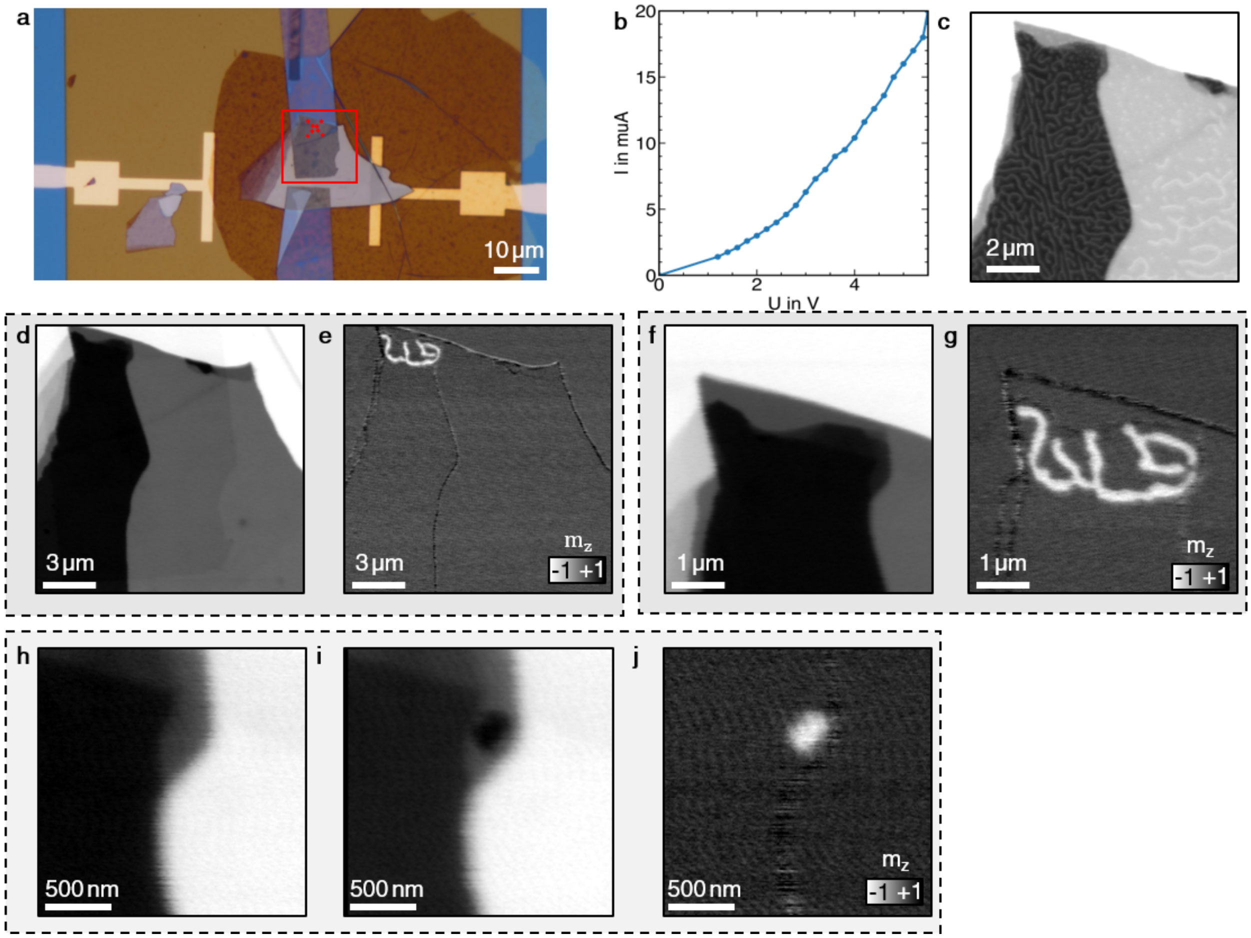

Supplementary Fig. S3: Second sample reproducing nanocontact formation and single skyrmion nucleation. (**a**) Optical image of the second sample. (**b**) Room temperature I-V curve of the created nanocontact. (**c**) Nucleation of magnetic skyrmions next to the nanocontact, and stripes far away from the contact at 160 K with 5.4 V and 20 mT. (**d**) STXM overview image of the investigated sample area. (**e**) STXM image after nanocontact creation (background was subtracted, inverted image). The magnetic stripes in the upper left corner were nucleated by applying a voltage of 1 V at 150 K and 0 mT. (**f**, **g**) Zoom into the created magnetic structure in panel b. (**h**) Saturated STXM image of the region comprising the nanocontact. (**i**) STXM image of a single skyrmion created at 150 K with 6 V and −25 mT. Note that the magnetic texture is opposite to that in panel c due to the inverse magnetic field. (**j**) STXM image of single skyrmion (background was subtracted). The polarization of the x-rays was c^+ for all images.

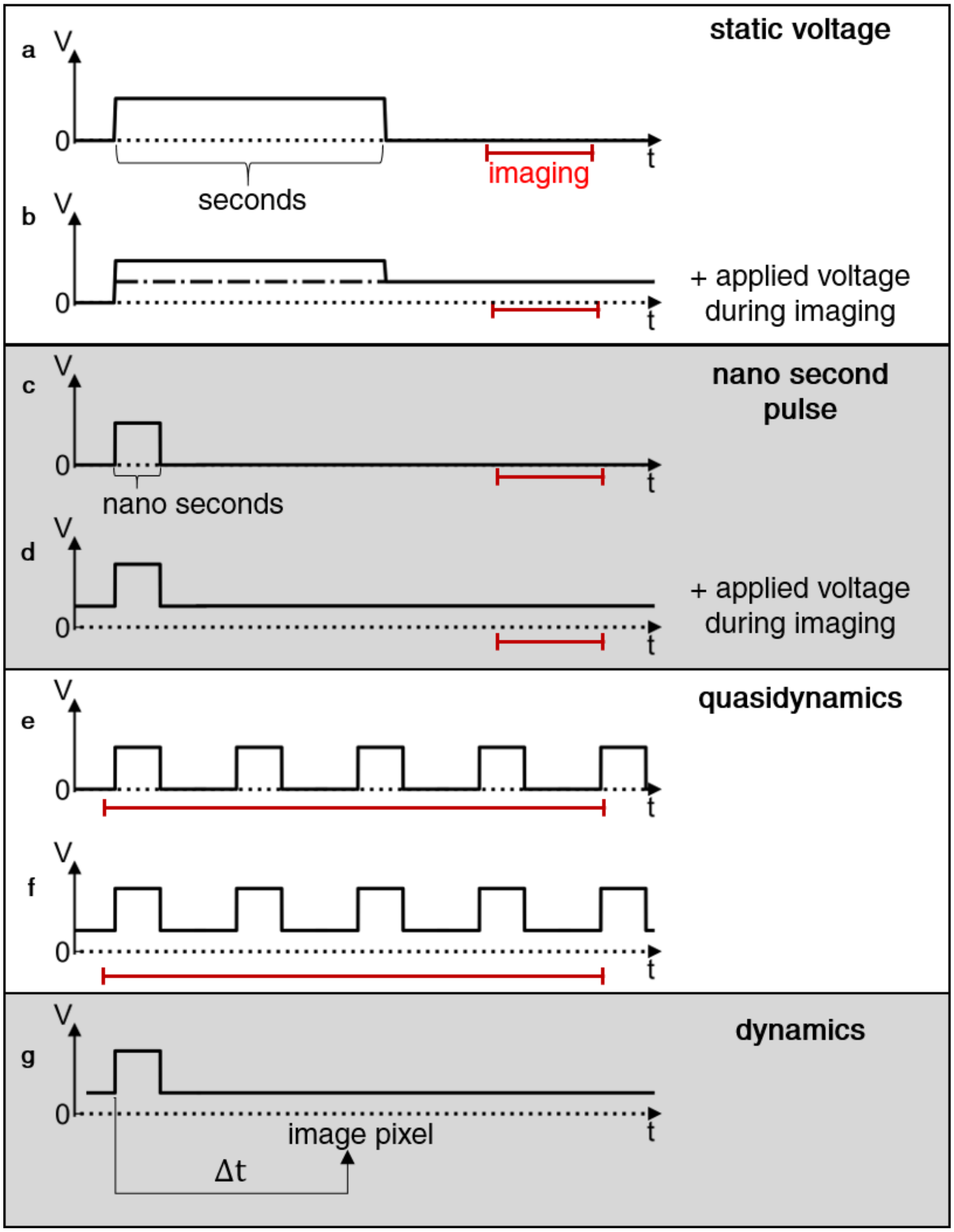

Supplementary Fig. S4: Overview of all used electrical manipulation and imaging protocols. (**a**) After applying a static voltage for a couple of seconds, the voltage is turned to 0 V, followed by imaging. (**b**) After applying a static voltage for a couple of seconds, the voltage is changed to a second static voltage and imaging is done while applying the voltage. (**c**) Applying a single nanosecond pulse and subsequent imaging with no voltage applied. (**d**)Applying a single nanosecond pulse and subsequent imaging while applying a voltage. (**e**) Applying a train of nanosecond pulses with time spacing of microseconds while imaging with an acquisition time of the order of 10 ms.(**f**) Same as in e), but including a static voltage offset. (**g**) Dynamical measurement in a stroboscopic pump-probe manner.

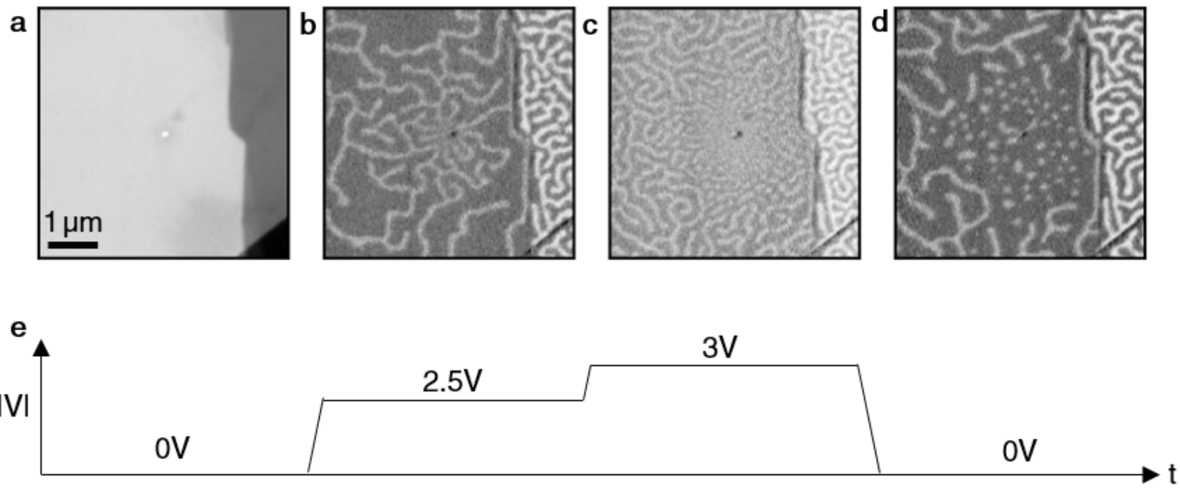

Supplementary Fig. S5: Exemplary series of steps to generate skyrmions using field cooling. a) STXM image taken at 0 V. STXM images with subtracted background acquired during the application of a DC voltage of b) -2.5 V, c) -3 V and d) 0 V. The polarization of the x-rays was c^{+}. The image contrast was inverted for better visibility. The magnetic field was $-10\,\mathrm{mT}$.

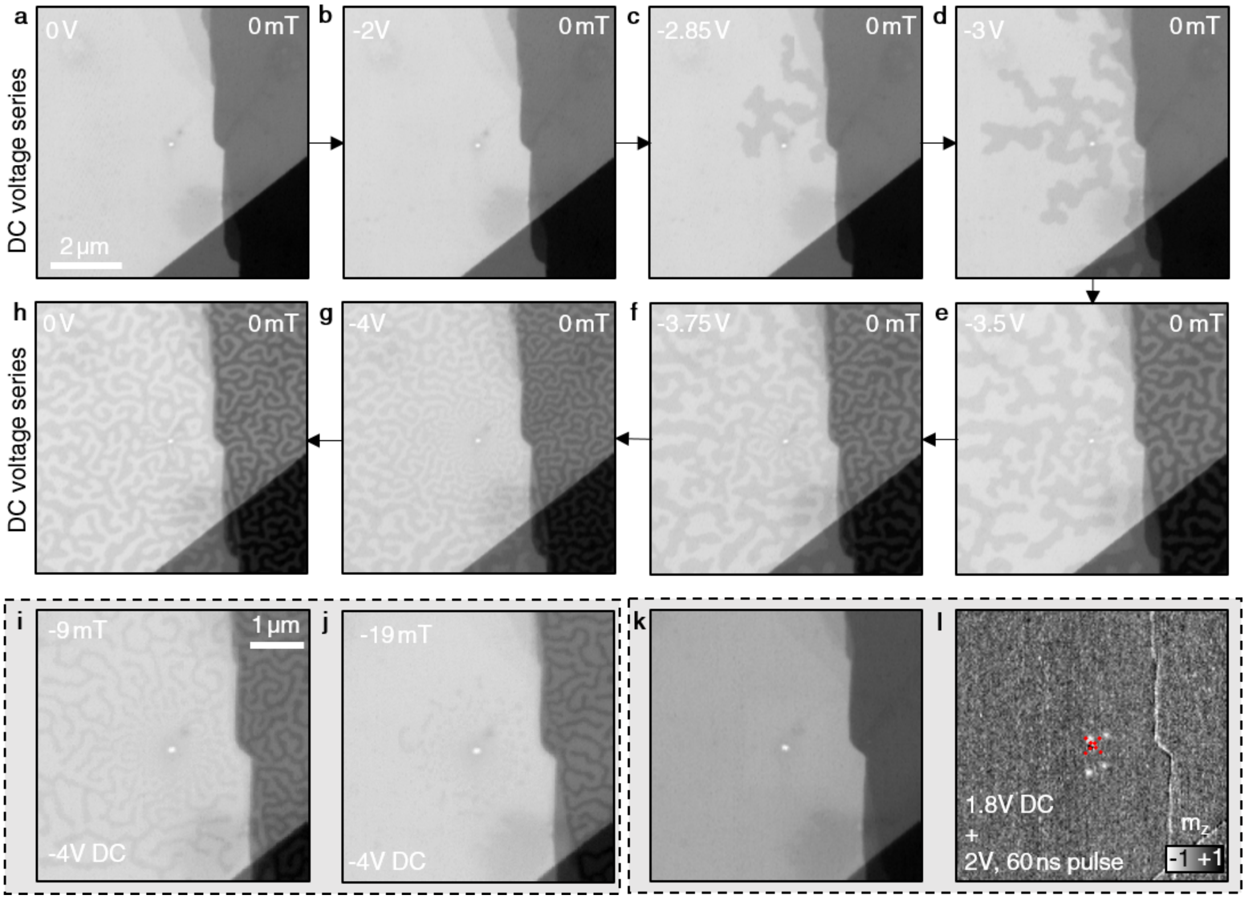

Supplementary Fig. S6: Influence of a DC current on the sample at 0 mT while imaging, and control of the spatial extent of nucleated magnetic structures. (**a**-**g**) Evolution of the magnetic state in dependence of an applied DC voltage. (**h**) Magnetic structure formed after decreasing the voltage to 0 V. The temperature was 150 K. (**i,j**) Magnetic texture while applying -4 V at (**i**) -9 mT and (**j**) -19 mT. (**k**) Saturated background STXM image for the same area. (**l**) STXM image with saturated background showing skyrmions generated at 150 K with a pulse height of 2 V and duration of 60 ns, and a DC offset voltage of 1.8 V. The red-dotted cross marks the position of the nanocontact. It can be seen that the size of the nucleation zone can be significantly reduced through suitable parameters. The polarization of the x-rays was c^+.

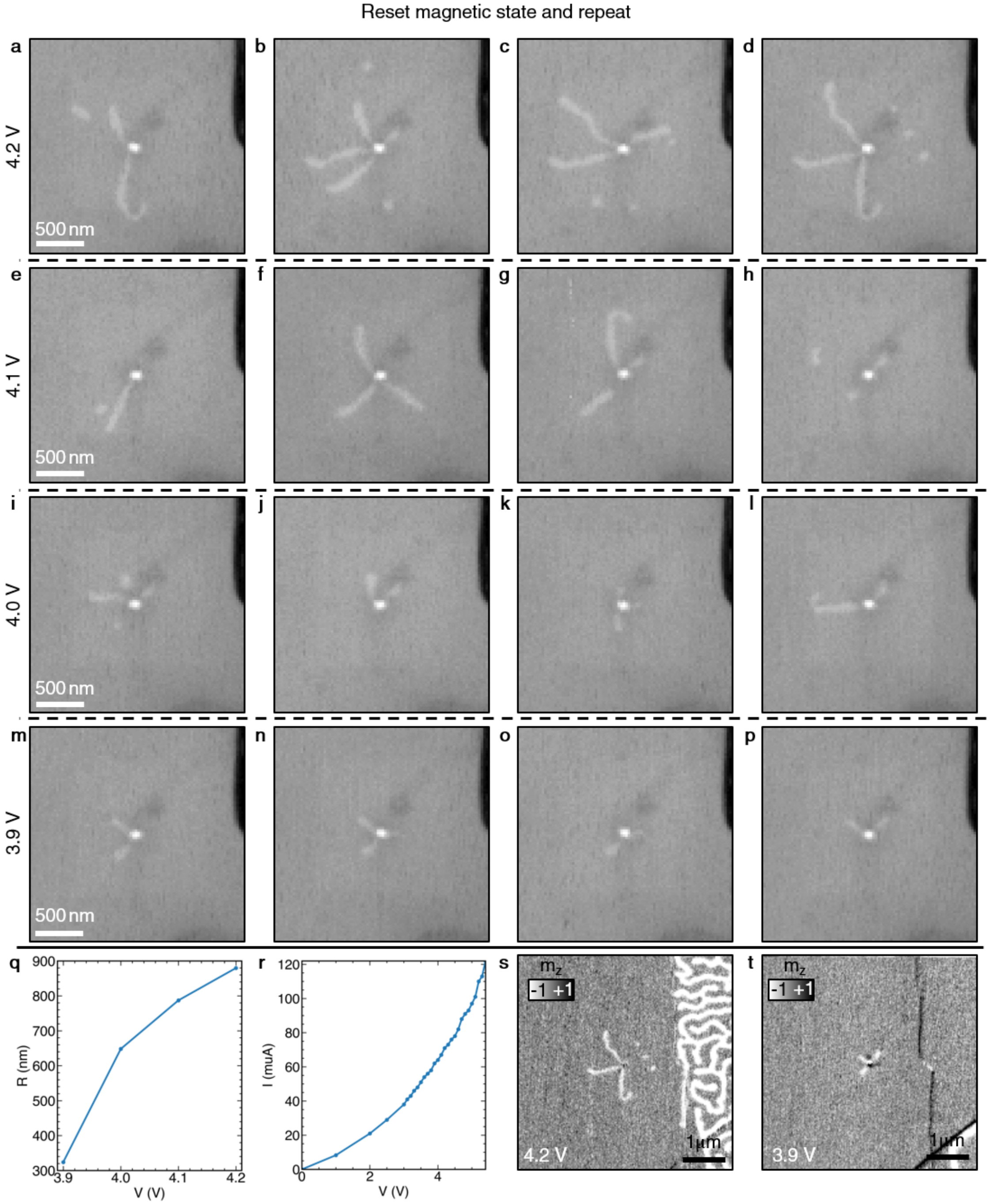

Supplementary Fig. S7: Extended data of magnetic stripe and skyrmion nucleation using a DC voltage series of $2.2\,\mathrm{V} \rightarrow V_{\mathrm{high}} \rightarrow 2.2\,\mathrm{V}$, with (**a-d**) $V_{\mathrm{high}} = 4.2\,\mathrm{V}$, (**e-h**) $V_{\mathrm{high}} = 4.1\,\mathrm{V}$, (**i-l**) $V_{\mathrm{high}} = 4.0\,\mathrm{V}$, and (**m-p**) $V_{\mathrm{high}} = 3.9\,\mathrm{V}$. (**q**) Maximal nucleation radius vs. V_{high}. In all cases, raw STXM data without background subtraction are shown. (**r**) Room temperature I-V curve of nanocontact. (**s**,**t**) Larger STXM scans around the above areas (background subtracted). All data were taken at 150 K under an applied field of $-16\,\mathrm{mT}$. The polarization of the x-rays was c^{-}.

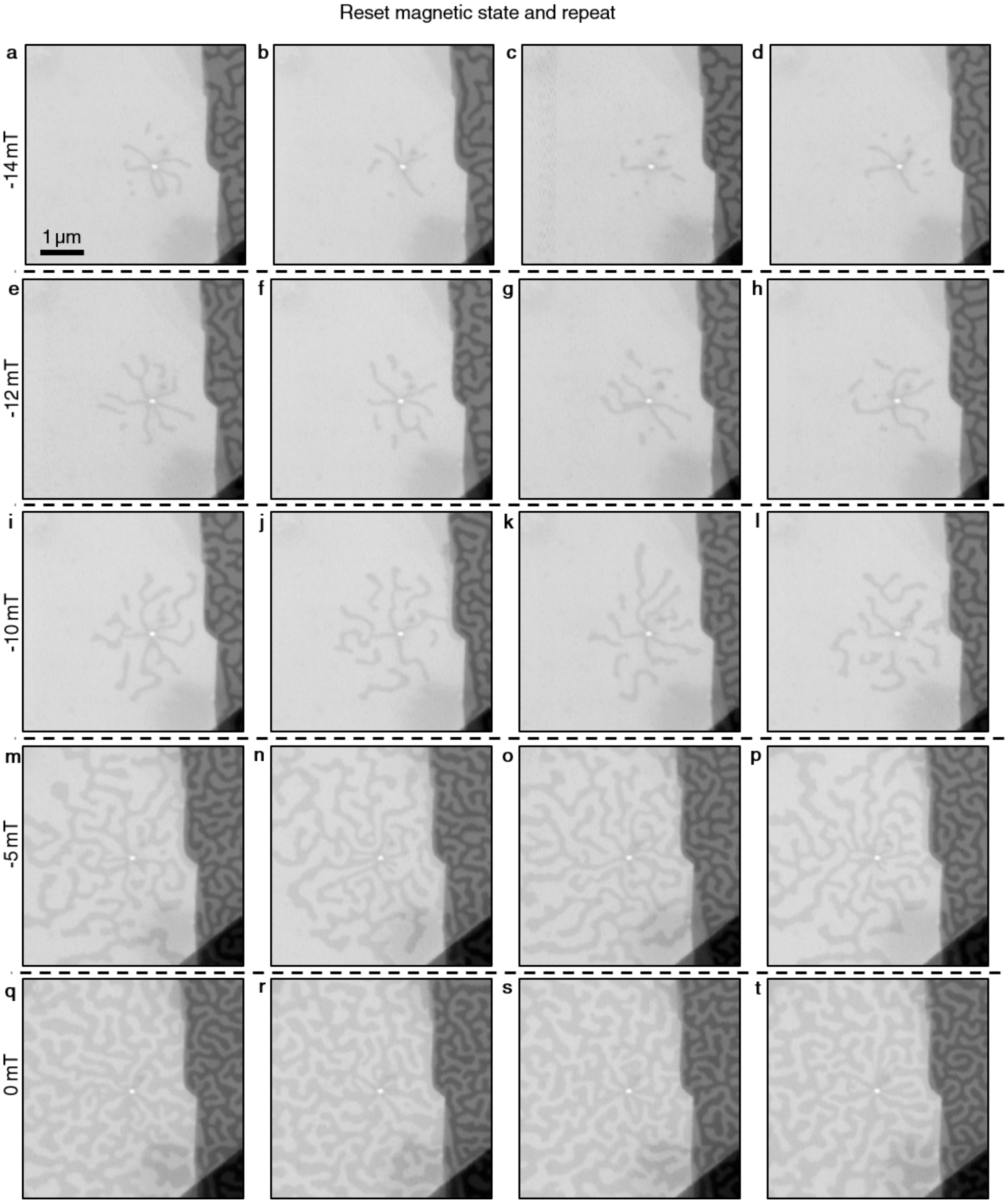

Supplementary Fig. S8: Overview of spin textures formed at different magnetic fields. The applied fields are (**a**- **d**) $-14\,\mathrm{mT}$, (**e-h**) $-12\,\mathrm{mT}$, (**i-l**) $-10\,\mathrm{mT}$, (**m-p**) $-5\,\mathrm{mT}$, and (**q-t**) $0\,\mathrm{mT}$ All data were taken at $150\,\mathrm{K}$. The protocol to induce and detect the spin textures involved the following steps: i) saturate the magnetic field at $-250\,\mathrm{mT}$, ii) apply the desired magnetic field, iii) apply a DC voltage of $-3.9\,\mathrm{V}$ for a few seconds, iv) apply a DC voltage of $-2\,\mathrm{V}$, and v) acquire STXM image. Note that the polarization in these images is opposite to the ones shown so far, which explains why the textures appear as black rather than white features. No background subtraction was done for all the images. The gray scale color depicts the out-of-plane magnetic contrast and the structural contrast. The polarization of the x-rays was c^{+}.

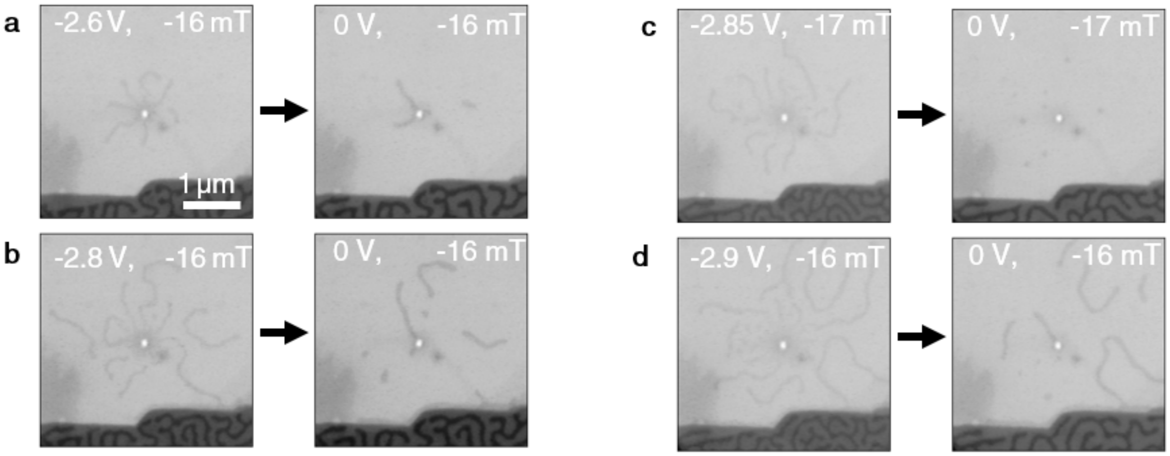

Supplementary Fig. S9: Effect of turning the applied static voltage to 0 V on the radially arranged stripe domains. For all four different samples (**a**-**d**) the stripes are seen to break up into smaller stripes and skyrmions.

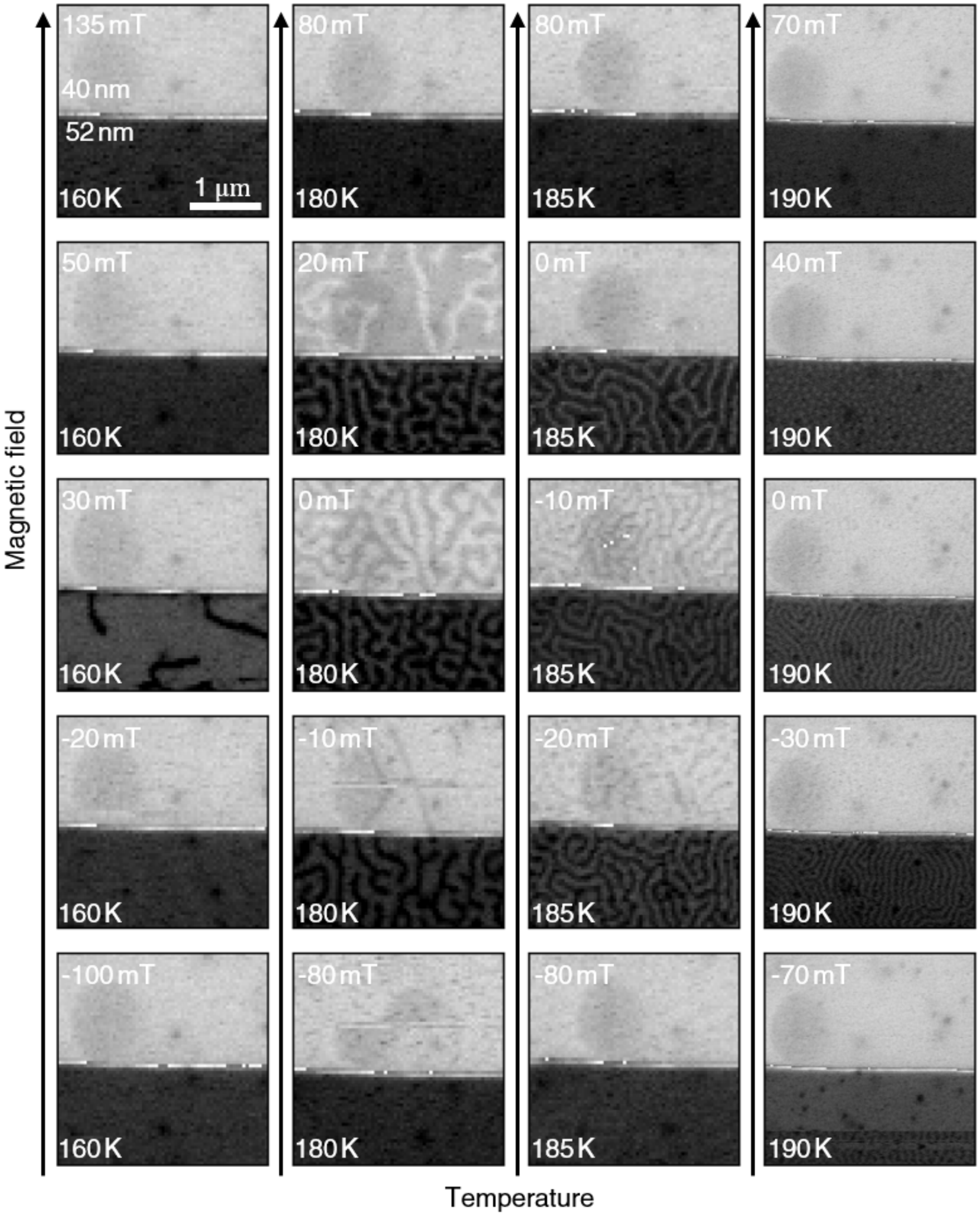

Supplementary Fig. S10: Examples of raw STXM images used to derive the phase diagram in main Fig. 2a. Each image is labeled with the corresponding magnetic field and temperature. The field protocol started with $-250\,\mathrm{mT}$, followed by ramping up the field in $10\,\mathrm{mT}$ steps until uniform magnetization is reached.

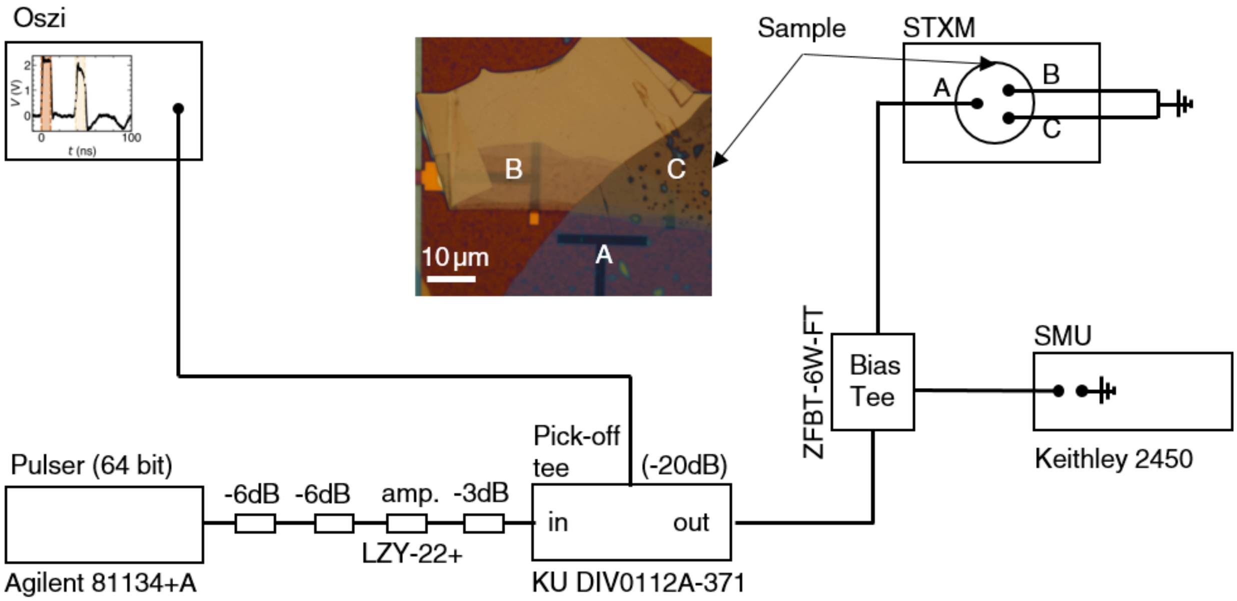

Supplementary Fig. S11: Equivalent circuit diagram and optical image of the device. The AC pulse originating from the pulser is amplified. The pick-off tee splits the AC signal, which enables monitoring the pulses send to the sample. A bias tee is used in order to add the DC signal sourced by a Keithley 2400 and the pulse. The electrical signal is send to the electrode A on the graphite. B and C connect FGT to GND. The optical image of the sample features three gold contacts A, B and C. The FGT sheet is contacted by contacts B and C. An hBN sheet serves to isolate the upper graphite electrode from the FGT sheet.

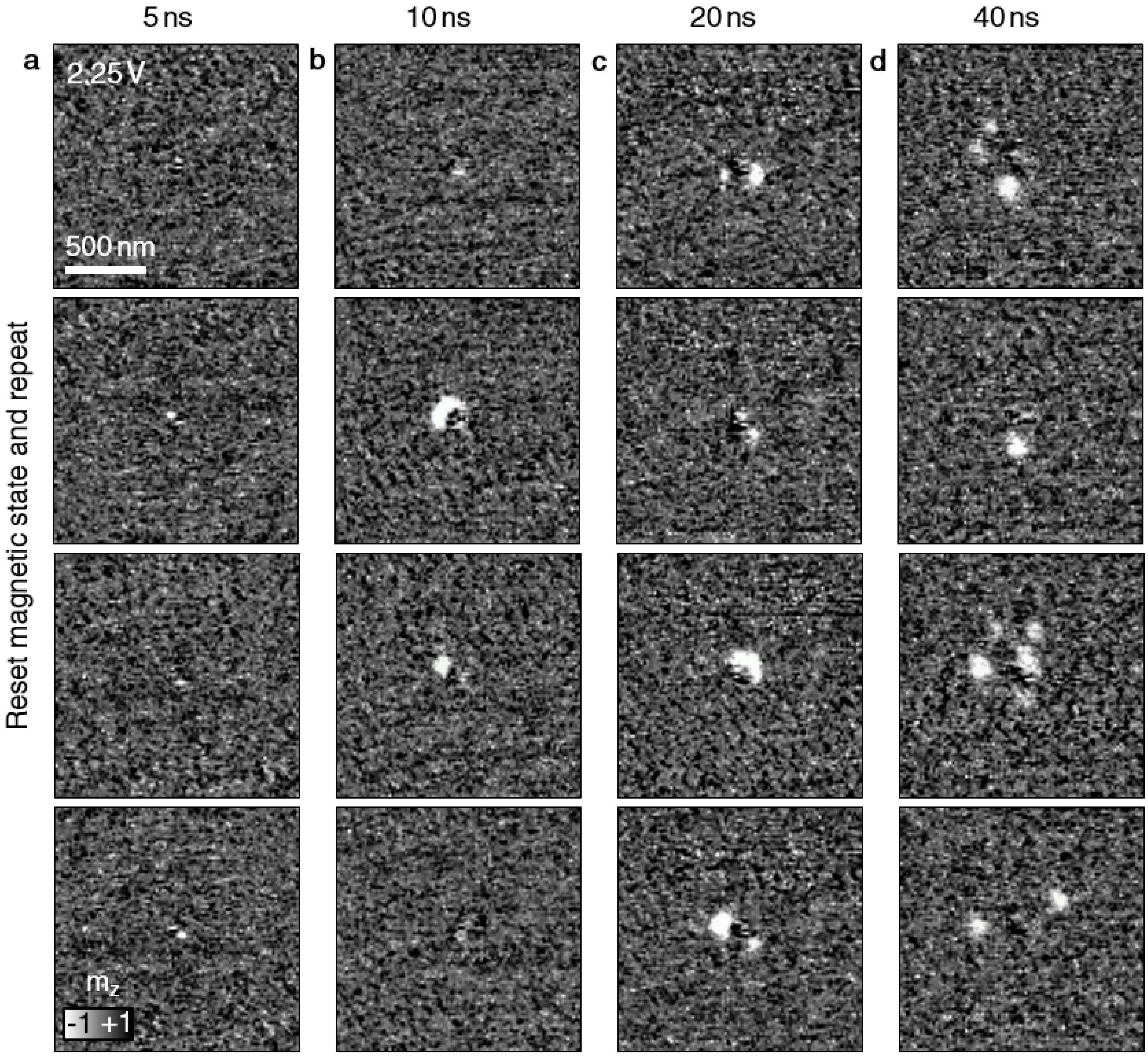

Supplementary Fig. S12: Extended data for pulsed skyrmion nucleation diagram. STXM images recorded after applying an electrical pulse with height of 2.25 V and varying pulse length between Δt =5 ns and 40 ns, as indicated. The pulse was applied to a uniform negative magnetised state at 160 K and −16 mT. Data used to construct main Fig. 3. The polarization of the x-rays was c^{-}.

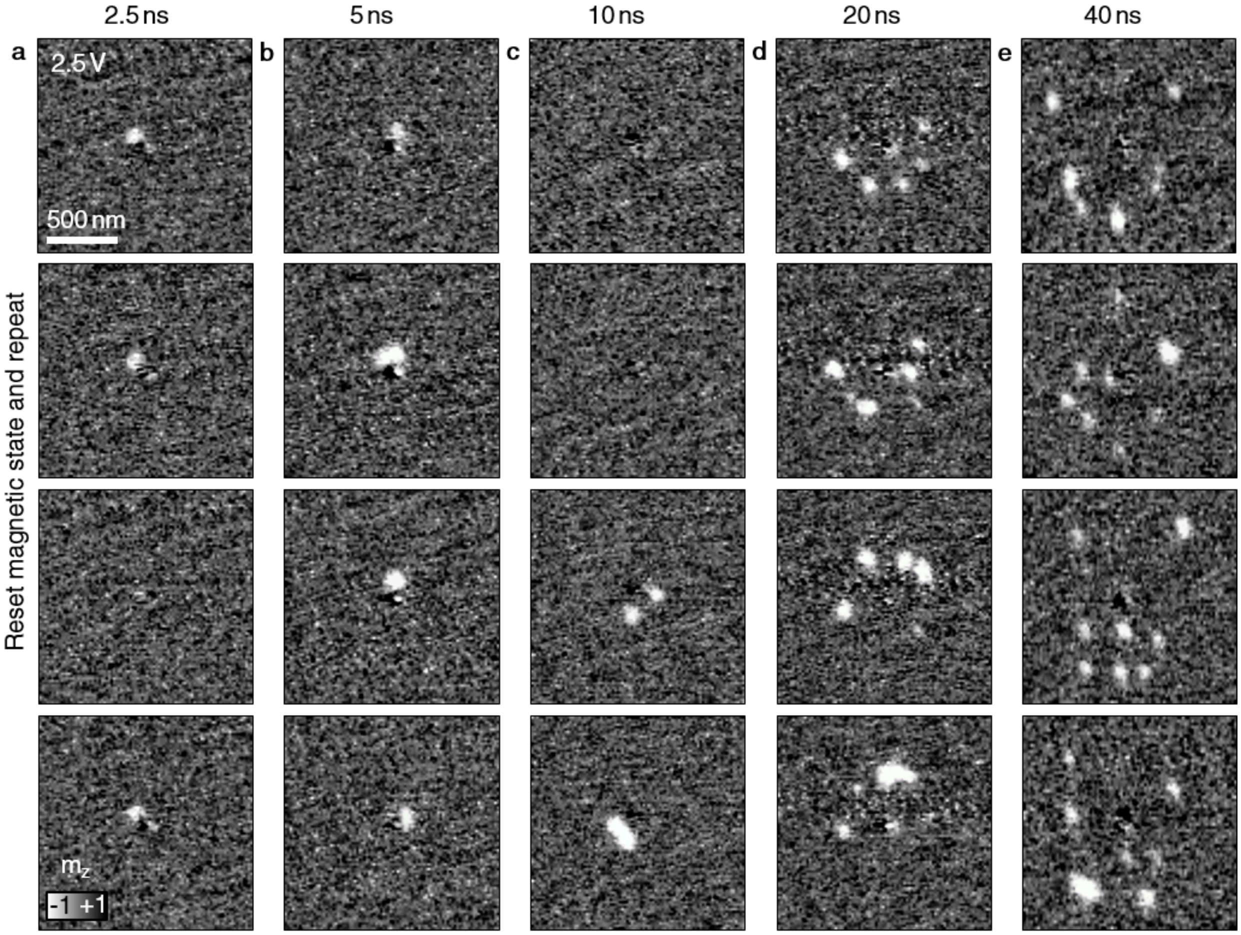

Supplementary Fig. S13: Extended data for pulsed skyrmion nucleation phase diagram. STXM images recorded after applying an electrical pulse with height of 2.5 V and varying pulse length between Δt =5 ns and 40 ns, as indicated. The pulse was applied to a uniform negative magnetised state at 160 K and −16 mT. Data used to construct main Fig. 3. The polarization of the x-rays was c^{-}.

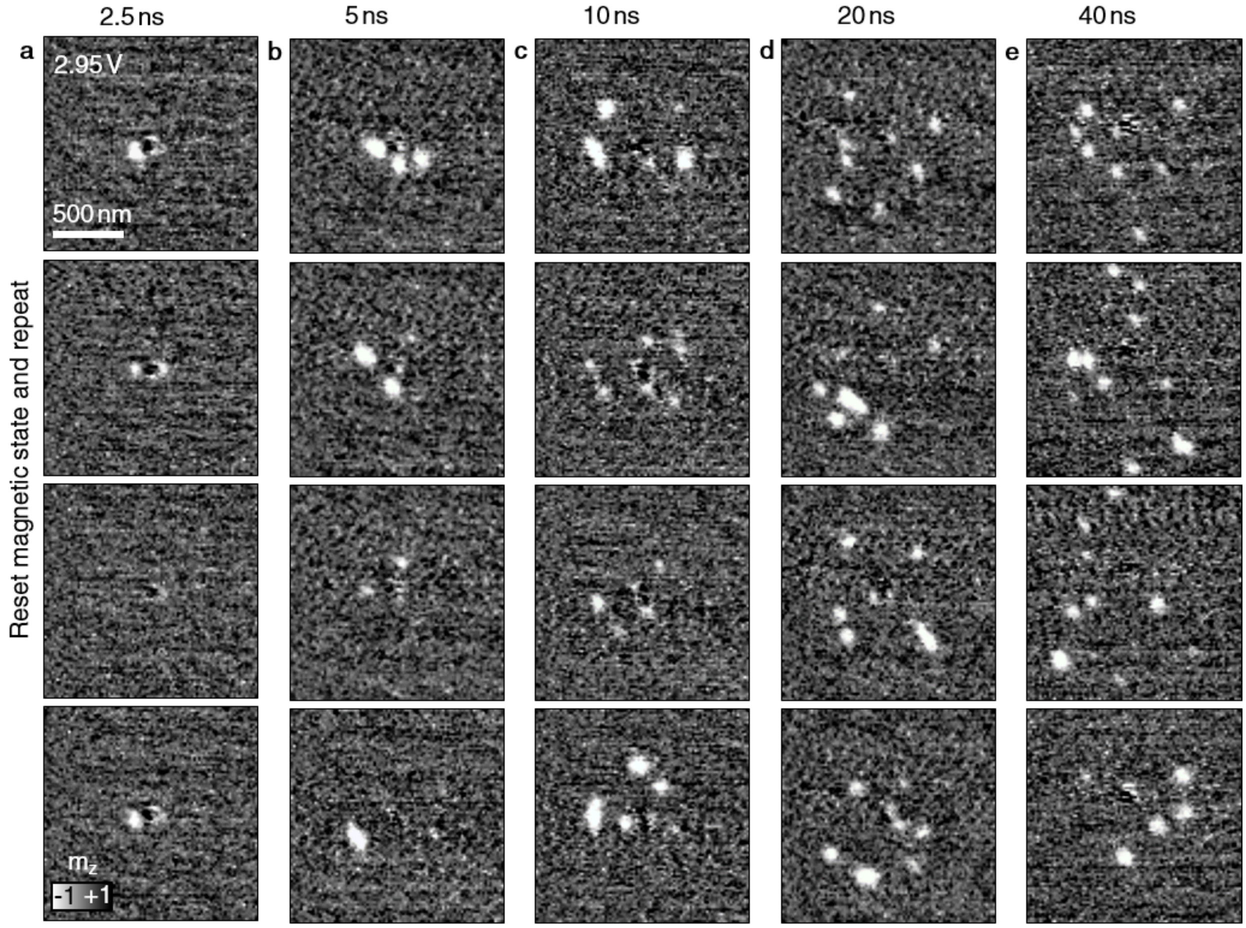

Supplementary Fig. S14: Extended data for pulsed skyrmion nucleation phase diagram. STXM images recorded after applying an electrical pulse with height of 2.95 V and varying pulse length between $\Delta t = 5$ ns and 40 ns, as indicated. The pulse was applied to a uniform negative magnetised state at 160 K and -16 mT. Data used to construct main Fig. 3. The polarization of the x-rays was c^{-}.

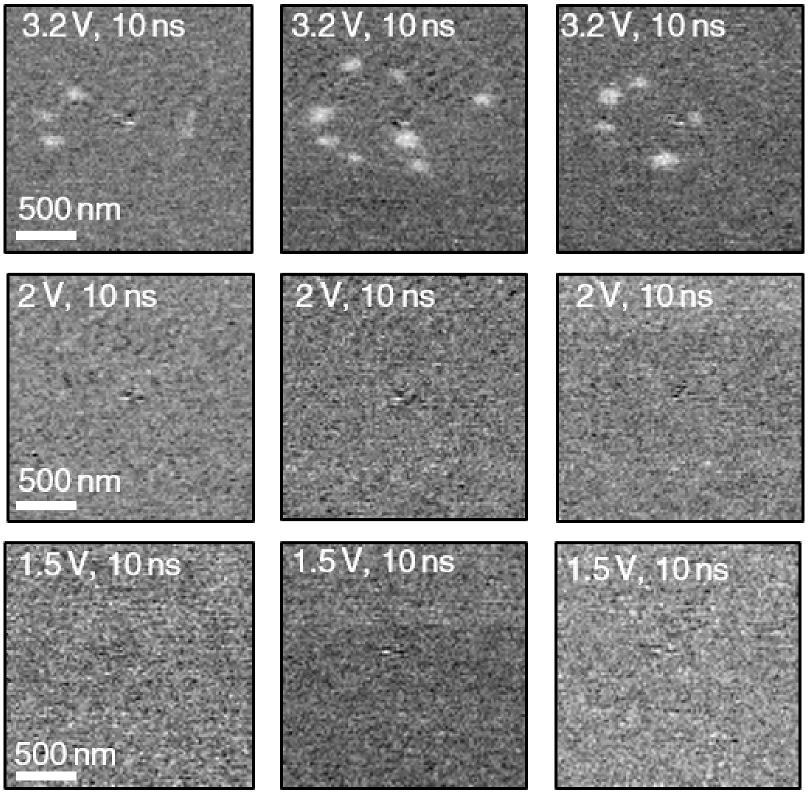

Supplementary Fig. S15: Extended data for pulsed skyrmion nucleation phase diagram. STXM images with subtracted background recorded after applying an electrical pulse with height indicated in the images. The pulse was applied to a uniform negative magnetised state at 160 K and −16 mT. Data used to construct main Fig. 3. The polarization of the x-rays was c^{-}.

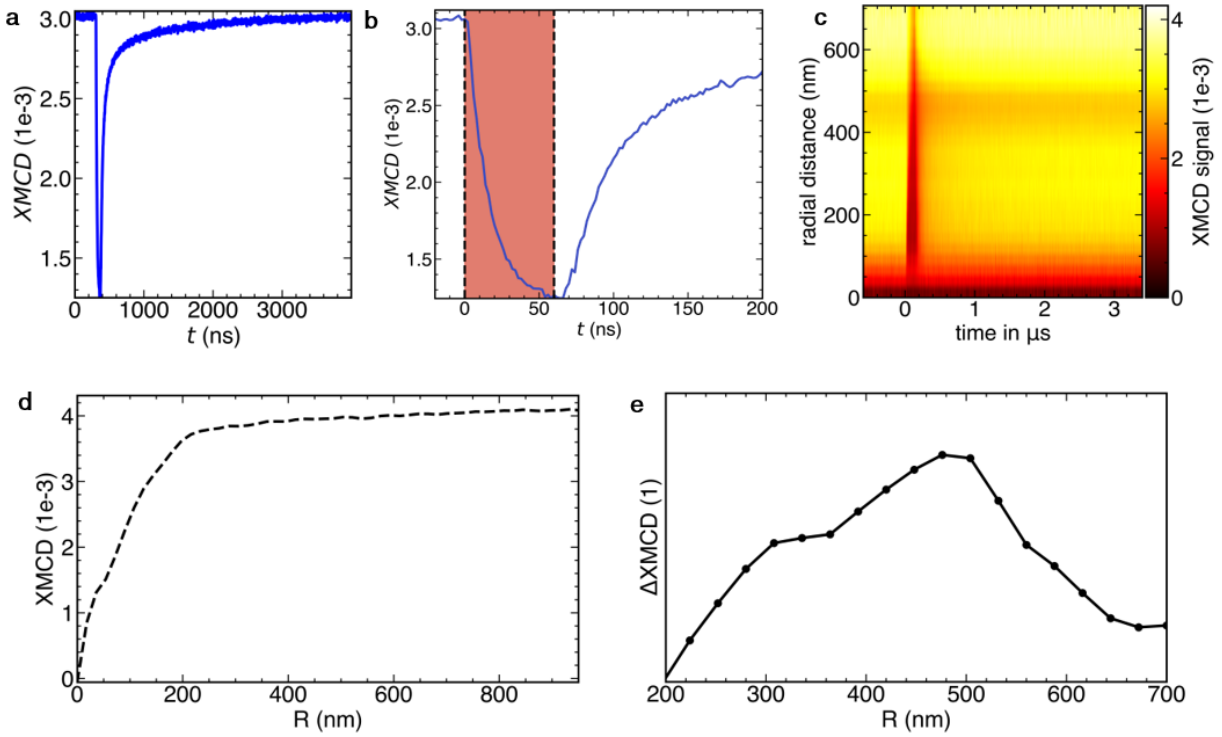

Supplementary Fig. S16: Extended data of the time-resolved measurements. (**a**) Time evolution of the average XMCD signal between a radius of 200 nm and 500 nm. (**b**) Zoom into the plot in panel a. The pulse duration (60 ns) is marked by the red rectangle. (**c**) Overview over the radially averaged XMCD signal for all measured times. (**d**) Plot of background magnetization as a function of radial distance. (**e**) The difference in XMCD signal between the time average of thermal equilibrium and background magnetisation could be interpreted as skyrmion nucleation probability, calculated based upon the difference.

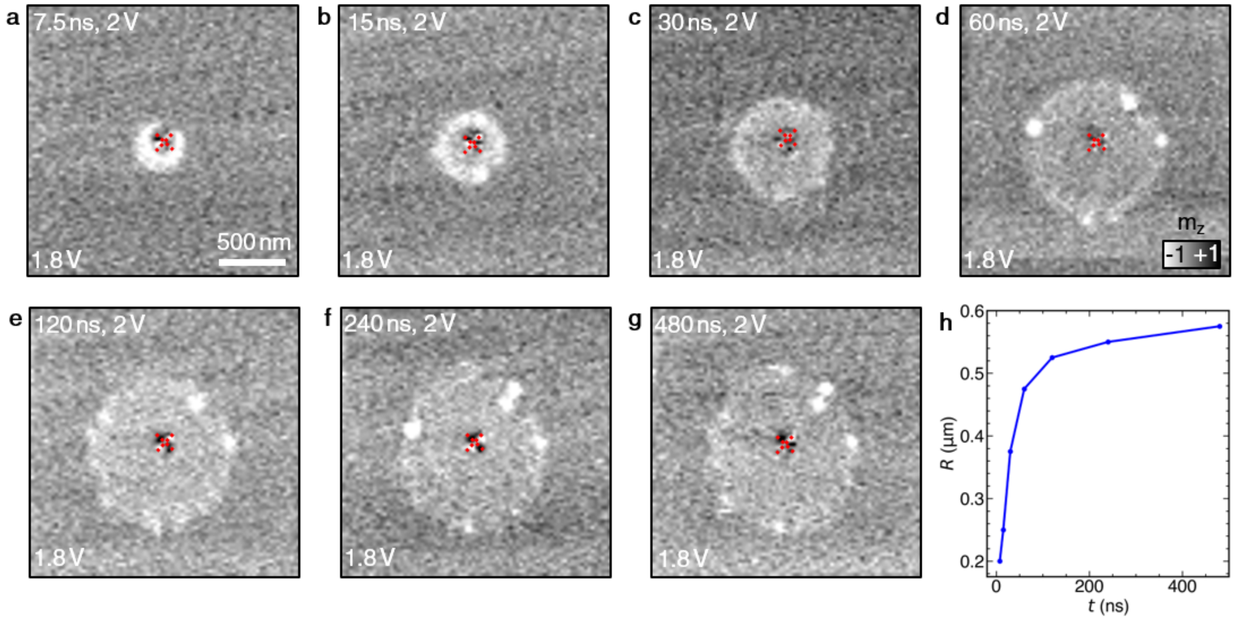

Supplementary Fig. S17: Quasidynamic images of halo for different pulse lengths. Quasidynamic means that for each pixel, we averaged over multiple pulses (depending on the acquisiton time and repetition rate). All images were taken at 150 K with a DC offset voltage of 1.8 V. The red dotted cross marks the position of the nanocontact. (**a-g**) STXM images with subtracted contrast in dependence of pulsing time. The pulse height was 2 V in all cases. The acquisition time per pixel was 10 ms, and the repetition rate (**a-e**) 3.84 µs, (**f**) 7.68 µs, and (**g**) 15.36 µs.(**h**) Dependence of halo radius on pulsing time, as extracted from the STXM images. The polarization of the x-rays was c^{-} and the applied magnetic field -16 mT.

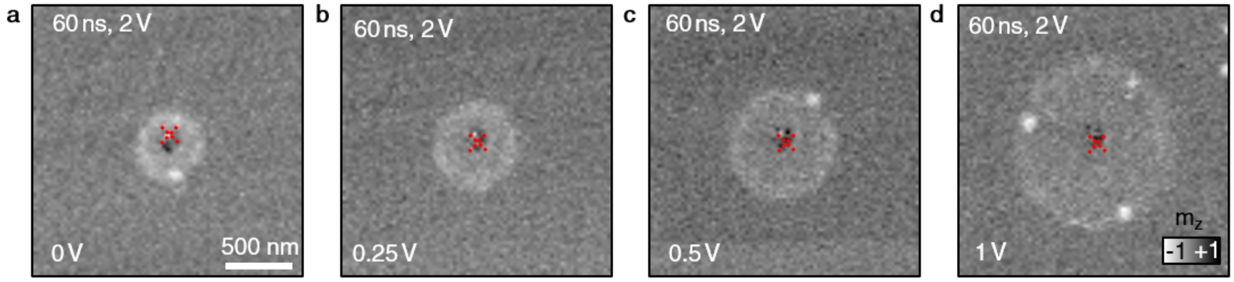

Supplementary Fig. S18: Quasidynamic images of halo in dependence of the applied DC offset. Quasidynamic means that for each pixel, we averaged over multiple pulses (depending on the acquisiton time and repetition rate). The acquisition time per pixel was 10 ms. (**a-d**) STXM images with subtracted background in dependence of the DC offset voltage. The red cross marks the position of the nanocontact. The data was taken at 150 K with a repetition rate of 3.84 µs, a pulse height of 2 V and a pulse length of 60 ns. The polarization of the x-rays was c^{-} and the applied magnetic field -16 mT.

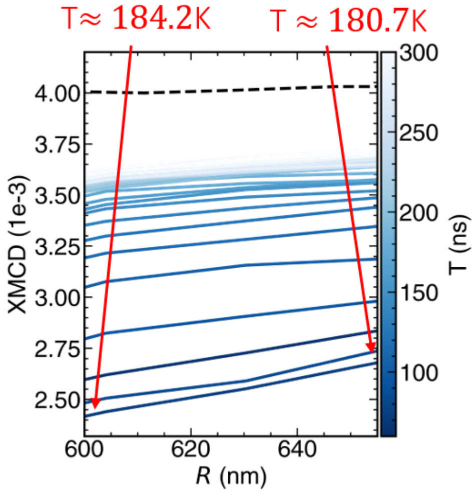

Supplementary Fig. S19: Temperature approximation based on the XMCD signal of the dynamical measurements away from the radius of skyrmion nucleation. The exponents for the temperature dependence of the magnetization were fitted to the experimental data following the approach described in reference.[6]

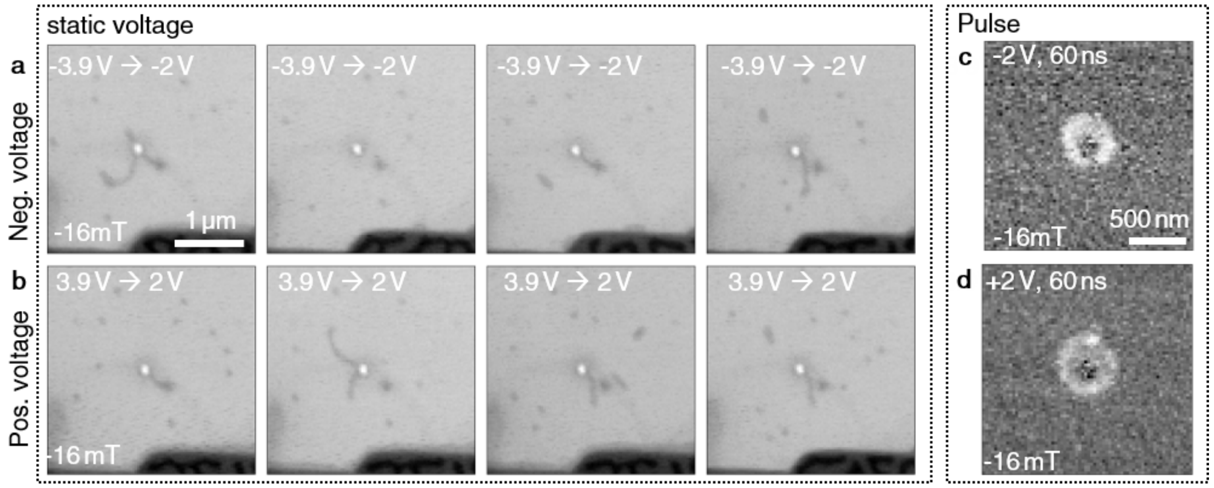

Supplementary Fig. S20: Voltage polarity dependence of the spin texture nucleation. Generation of skyrmions with (a) a negative DC voltage and (b) a positive DC voltage. In both cases, the polarization of the x-rays was c^+, and the applied magnetic field -16 mT. c,d) Quasidynamic images of the same nanocontact, recorded with an acquisition time for each pixel of 10 ms, a pulse length of 60 ns, a repetition rate of 3.84 µs, and a voltage pulse height of (c) -2 V and (d) 2 V. The polarization of the x-rays for was c^-, and the applied magnetic field -16 mT.

References

(1) Deiseroth, H.-J.; Aleksandrov, K.; Reiner, C.; Kienle, L.; Kremer, R. K. Fe3GeTe2 and Ni3GeTe2–Two New Layered Transition-Metal Compounds: Crystal Structures, HRTEM Investigations, and Magnetic and Electrical Properties. 2006.

(2) Chen, B.; Yang, J.; Wang, H.; Imai, M.; Ohta, H.; Michioka, C.; Yoshimura, K.; Fang, M. Magnetic properties of layered itinerant electron ferromagnet Fe3GeTe2. *Journal of the Physical Society of Japan* **2013**, *82*, 124711.

(3) Lemesh, I.; Litzius, K.; Böttcher, M.; Bassirian, P.; Kerber, N.; Heinze, D.; Zázvorka, J.; Büttner, F.; Caretta, L.; Mann, M., et al. Current-Induced Skyrmion Generation through Morphological Thermal Transitions in Chiral Ferromagnetic Heterostructures. *Advanced materials* **2018**, *30*, 1805461.

(4) Gilmore, R.; Besser, L. Practical RF circuit design for modern wireless systems. *Artech House* **2003**, *2*, S480.

(5) Litzius, K. et al. The role of temperature and drive current in skyrmion dynamics. *Nature Electronics* **2020**, *3*, 30–36.

(6) Evans, R. F.; Atxitia, U.; Chantrell, R. W. Quantitative simulation of temperature-dependent magnetization dynamics and equilibrium properties of elemental ferromagnets. *Physical Review B* **2015**, *91*, 144425.
